# Time-lapse imaging of microRNA activity reveals the kinetics of microRNA activation in single living cells

**DOI:** 10.1038/s41598-017-12879-2

**Published:** 2017-10-03

**Authors:** Hideaki Ando, Matsumi Hirose, Gen Kurosawa, Soren Impey, Katsuhiko Mikoshiba

**Affiliations:** 1 Laboratory for Developmental Neurobiology, RIKEN Brain Science Institute, Wako, Saitama, 351-0198 Japan; 2 Theoretical Biology Laboratory, RIKEN, Wako, Saitama, 351-0198 Japan; 3 Oregon Stem Cell Center, Oregon Health and Science University, Portland, OR 97239 USA

## Abstract

MicroRNAs (miRNAs) are small, non-coding RNAs that play critical roles in the post-transcriptional regulation of gene expression. Although the molecular mechanisms of the biogenesis and activation of miRNA have been extensively studied, the details of their kinetics within individual living cells remain largely unknown. We developed a novel method for time-lapse imaging of the rapid dynamics of miRNA activity in living cells using destabilized fluorescent proteins (dsFPs). Real-time monitoring of dsFP-based miRNA sensors revealed the duration necessary for miRNA biogenesis to occur, from primary miRNA transcription to mature miRNA activation, at single-cell resolution. Mathematical modeling, which included the decay kinetics of the fluorescence of the miRNA sensors, demonstrated that miRNAs induce translational repression depending on their complementarity with targets. We also developed a dual-color imaging system, and demonstrated that miR-9-5p and miR-9-3p were produced and activated from a common hairpin precursor with similar kinetics, in single cells. Furthermore, a dsFP-based miR-132 sensor revealed the rapid kinetics of miR-132 activation in cortical neurons under physiological conditions. The timescale of miRNA biogenesis and activation is much shorter than the median half-lives of the proteome, suggesting that the degradation rates of miRNA target proteins are the dominant rate-limiting factors for miRNA-mediated gene silencing.

## Introduction

MicroRNAs (miRNAs) are a large family of small, non-coding RNAs that play critical roles in the post-transcriptional regulation of gene expression. MiRNAs are predicted to regulate more than half of all mammalian protein-coding genes, and are involved in almost all developmental and cellular processes^1^. The canonical pathway of miRNA biogenesis in animals is initiated by transcription of long primary miRNAs (pri-miRNAs) by RNA polymerase II^2,3^. The pri-miRNAs are processed in the nucleus by Drosha (a class 2 ribonuclease III enzyme) into hairpin intermediates of approx. 70 nucleotides in length termed pre-miRNAs^4^. Pre-miRNAs are transported to the cytoplasm by exportin-5^5,6^, where they are further cleaved by Dicer (another RNase III enzyme) into approx. 22-bp duplex molecules with short 3′ overhangs^7–9^. One strand of the duplex, the guide strand, is selectively incorporated into the RNA-induced silencing complex (RISC) containing the Argonaute (Ago) protein. The other strand, the passenger strand, is discarded^10,11^. miRNAs bind to their target mRNAs by base pairing with partially complementary sequences in the 3′-untranslated region (3′ UTR). The specificity of target recognition is mainly determined by the seed sequence (nucleotide positions 2–7) of the miRNA strand^1^. Binding of miRNAs to target mRNAs results in translational repression and/or mRNA degradation^12^.

To understand the spatiotemporal dynamics of miRNA-mediated gene regulation, it is necessary to clarify the kinetics of miRNA biogenesis and activation within individual living cells. Expression levels of miRNA can be analyzed by northern blotting, quantitative PCR, microarrays, and deep sequencing; however, kinetic analysis is laborious due to the need to collect samples at multiple time points. Furthermore, these methods fail to capture information on cell-to-cell variations in miRNA expression that occur within individual cells. As a non-invasive imaging method, molecular beacons—which typically consist of stem-loop DNA oligonucleotides complementary to a miRNA strand, a fluorophore, and a quencher—overcome these limitations^13–16^. However, signals of molecular beacons arise from hybridization of mature miRNA to stem-loop DNA, regardless of Ago loading; thus, molecular beacons do not discriminate between Ago-loaded functional miRNA and free, nonfunctional miRNA. Because miRNA expression levels do not necessarily correlate with miRNA activity^17^, miRNA activity cannot be inferred from expression analysis alone. To directly measure miRNA activity, luciferase genes with miRNA target sequences in their 3′ UTR have been widely used as reporter assays, and are also successfully utilized for bioluminescent imaging *in vivo* ^15,18–20^. However, current applications of luciferase-based methods are limited to the visualization of organs and cell aggregates implanted in mice, and these methods have not been utilized for imaging of miRNA activity at a single-cell resolution.

Green fluorescent protein (GFP) is another reporter gene widely used to visualize miRNA activity in living cells and in animals^21–25^. However, compared to luciferases, in this context GFP is an excessively stable protein; its slow turnover rate is not appropriate for monitoring the rapid temporal dynamics of miRNA activity in the time scales anticipated for miRNA biogenesis. To reveal the kinetics of miRNA biogenesis and activation within individual living cells, we utilized destabilized fluorescent proteins (dsFPs), which have rapid turnover rates due to the addition of a protein degradation sequence (PEST sequence)^26^. We developed a novel method for time-lapse imaging of miRNA activity in single living cells by using dsFP-based miRNA sensors. We estimated the time necessary for miRNA biogenesis to occur, from initiation at pri-miRNA transcription to completion at mature miRNA activation, and determined that it was much faster than the median half-lives of proteins. These results suggest that the stability of miRNA target proteins have great impacts on the output of miRNA function, and also imply a preventive mechanism of miRNA-mediated gene regulation.

## Results

### Decay kinetics of dsGFP- and GFP-based miRNA sensors

**Figure 1:**
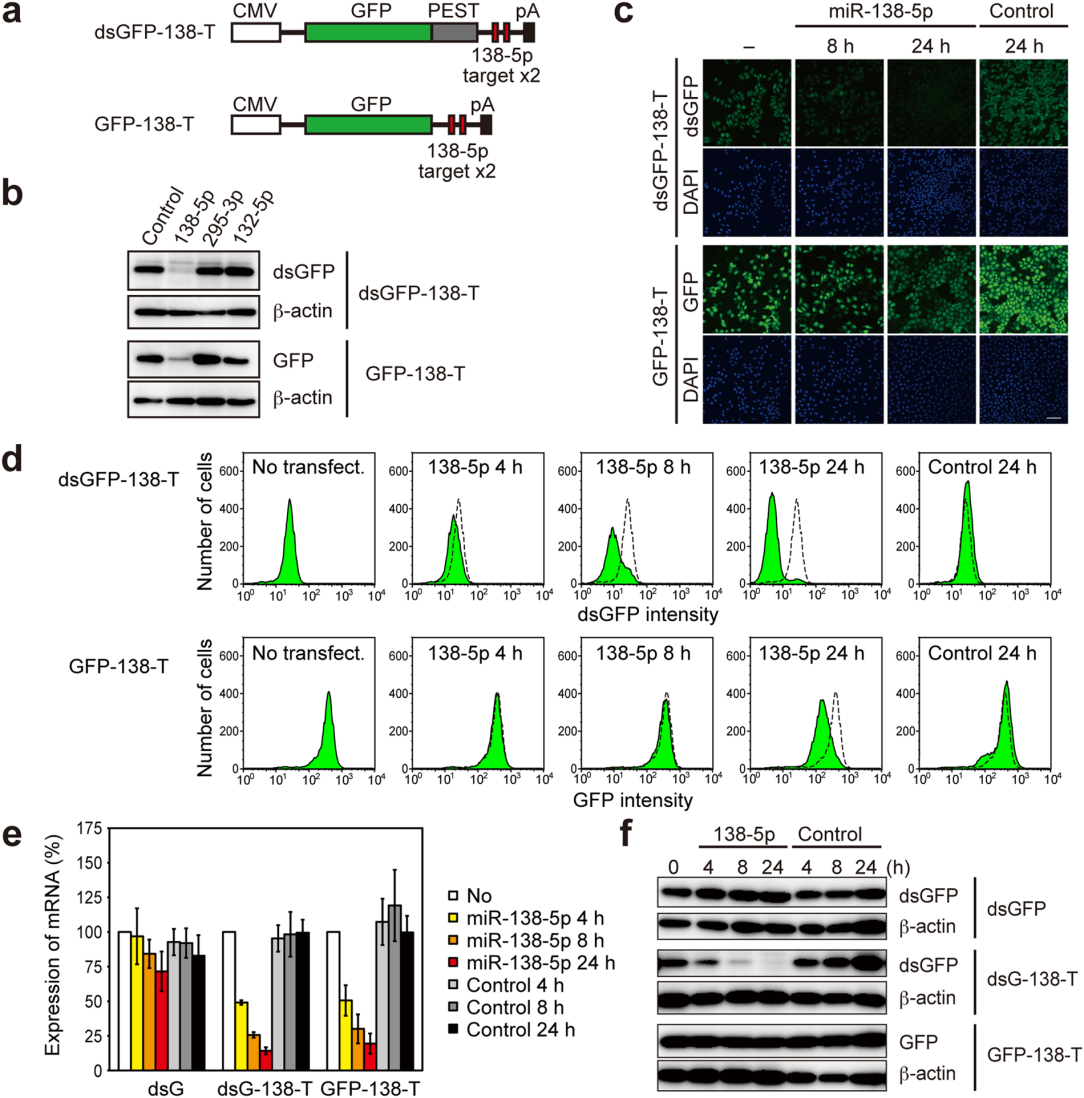
Rapid suppression of the dsGFP-based miRNA sensor. (**a**) Structure of miR-138-5p sensors. (**b**) Plasmids encoding dsGFP-138-T or GFP-138-T (0.5 µg) were co-transfected with 1 nM miR-138-5p, miR-295-3p, miR-132-3p, or control miRNA mimics into HeLa cells. 24 h after transfection, cells were analyzed by western blotting with anti-GFP and anti-β-actin antibodies. (**c**,**d**) Stable HeLa cells expressing dsGFP-138-T or GFP-138-T were transfected with 1 nM miR-138-5p or control miRNA mimics. Fluorescence of miRNA sensors was analyzed by confocal microscopy (**c**) or flow cytometry (**d**). Scale bars: 100 µm. (**e**) Stable HeLa cells expressing dsGFP, dsGFP-138-T, or GFP-138-T were transfected with 1 nM miR-138-5p or control miRNA mimics. mRNA expression levels of miRNA sensors were analyzed by quantitative RT-PCR. Data are expressed as mean ± SD (n = 3). (**f**) Stable HeLa cells transfected with miRNA mimics were analyzed by western blotting with anti-GFP and anti-β-actin antibodies.

The half-lives of GFP and destabilized GFP (dsGFP) in mammalian cells are ~26 h and ~2 h, respectively^26,27^. To analyze the impact of reporter protein stability on the output of miRNA-mediated gene regulation, we generated miRNA sensors using dsGFP or GFP driven by the CMV promoter sequence. Because the presence of two optimally spaced target sites increases the efficiency of miRNA function^28^, we inserted two perfect-match complementary sequences of a brain-enriched miRNA, miR-138-5p, into the 3′ UTR of dsGFP and of GFP; we termed these constructs dsGFP-138-T and GFP-138-T, respectively (Fig. 1a). We co-transfected plasmids encoding either dsGFP-138-T or GFP-138-T with synthetic miRNA mimics into HeLa cells and analyzed their expression 24 h after transfection. Both dsGFP-138-T and GFP-138-T were specifically suppressed by miR-138-5p, but not by other miRNAs (Fig. 1b). In a similar fashion, we also constructed dsGFP-295-T and GFP-295-T by inserting two perfect-match complementary sequences of an embryonic stem cell-specific miRNA, miR-295-3p, into the 3′ UTR of dsGFP and of GFP, respectively. We confirmed that expressions of dsGFP-295-T and GFP-295-T were specifically suppressed by co-transfection of miR-295-3p mimics (Supplementary Fig. S1).

To compare the decay kinetics of dsGFP-based and GFP-based miRNA sensors, we generated stable HeLa cell lines expressing dsGFP-138-T or GFP-138-T. These stable HeLa cells were transfected with miRNA mimics; 8 or 24 h after transfection, fluorescence from dsGFP or GFP in these cells was analyzed by confocal microscopy. As early as 8 h after transfection, dsGFP-138-T was efficiently suppressed by miR-138-5p (Fig. 1c). By contrast, suppression of GFP-138-T was slow to occur, with partial decreases in expression observed 24 h after transfection (Fig. 1c). Next, stable HeLa cells expressing miRNA sensors were transfected with miRNA mimics, and fluorescence levels were then analyzed by flow cytometry either 4, 8, or 24 h after transfection. Decreases in dsGFP-138-T fluorescence due to suppression by miR-138-5p, but not due to suppression by control miRNA, were detected as early as 4 h after transfection with miRNA mimics (Fig. 1d). By contrast, decrease in the fluorescence of GFP-138-T was not clearly observed at timepoints 4 h and 8 h after transfection, and were detected only at 24 h after transfection (Fig. 1d). Essentially the same results were obtained for miR-295-3p sensors (Supplementary Fig. S1). These results suggest that even after the translation processes for GFP-138-T and GFP-295-T were repressed by miR-138-5p and miR-295-3p, respectively, the fluorescence of pre-existing GFP-138-T and GFP-295-T still remained—due to the long half-life of GFP. By contrast, dsGFP-138-T and dsGFP-295-T quickly disappeared, because of the instability of dsGFP.

To confirm the aforementioned explanations, we analyzed the levels of mRNA and the protein levels of the miRNA sensors. Stable HeLa cells expressing either dsGFP- or GFP-based miRNA sensors were transfected with miRNA mimics; 4, 8, or 24 h after transfection, expression levels of mRNA and of miRNA sensor proteins were then analyzed by quantitative RT-RCR and western blotting, respectively. In a similar time course, the mRNAs of dsGFP-138-T and GFP-138-T were rapidly suppressed by the miR-138-5p mimics, but not by control RNA (Fig. 1e). Likewise, the mRNAs of dsGFP-295-T and GFP-295-T were suppressed by the miR-295-3p mimics in a kinetically comparable fashion (Supplementary Fig. S1). These results suggest that these miRNA mimics induced mRNA degradation for both the dsGFP-based and the GFP-based miRNA sensors, probably through mRNA cleavage in an Ago2-dependent manner^29–31^. At the protein level, however, even 24 h after transfection, suppression of GFP-138-T and GFP-295-T was hardly seen (Fig. 1f, Supplementary Fig. S1). Little suppression of GFP-based sensors was quite a contrast to their efficient suppression when plasmids encoding GFP-based sensors were transiently co-transfected with miRNA mimics (Fig. 1b, Supplementary Fig. S1). By contrast, the rapid downregulations of dsGFP-138-T and dsGFP-295-T by miR-138-5p and miR-295-3p, respectively, were observed (Fig. 1f, Supplementary Fig. S1). These results indicate that when miRNAs suppress pre-existing miRNA target proteins, the half-lives of target proteins markedly affect the outcome of miRNA action. Thus, high turnover rates for miRNA sensor proteins are a critical requirement for the detection of the rapid dynamics of miRNA activity.

### Time-lapse imaging of miR-138-5p activation in living cells

**Figure 2:**
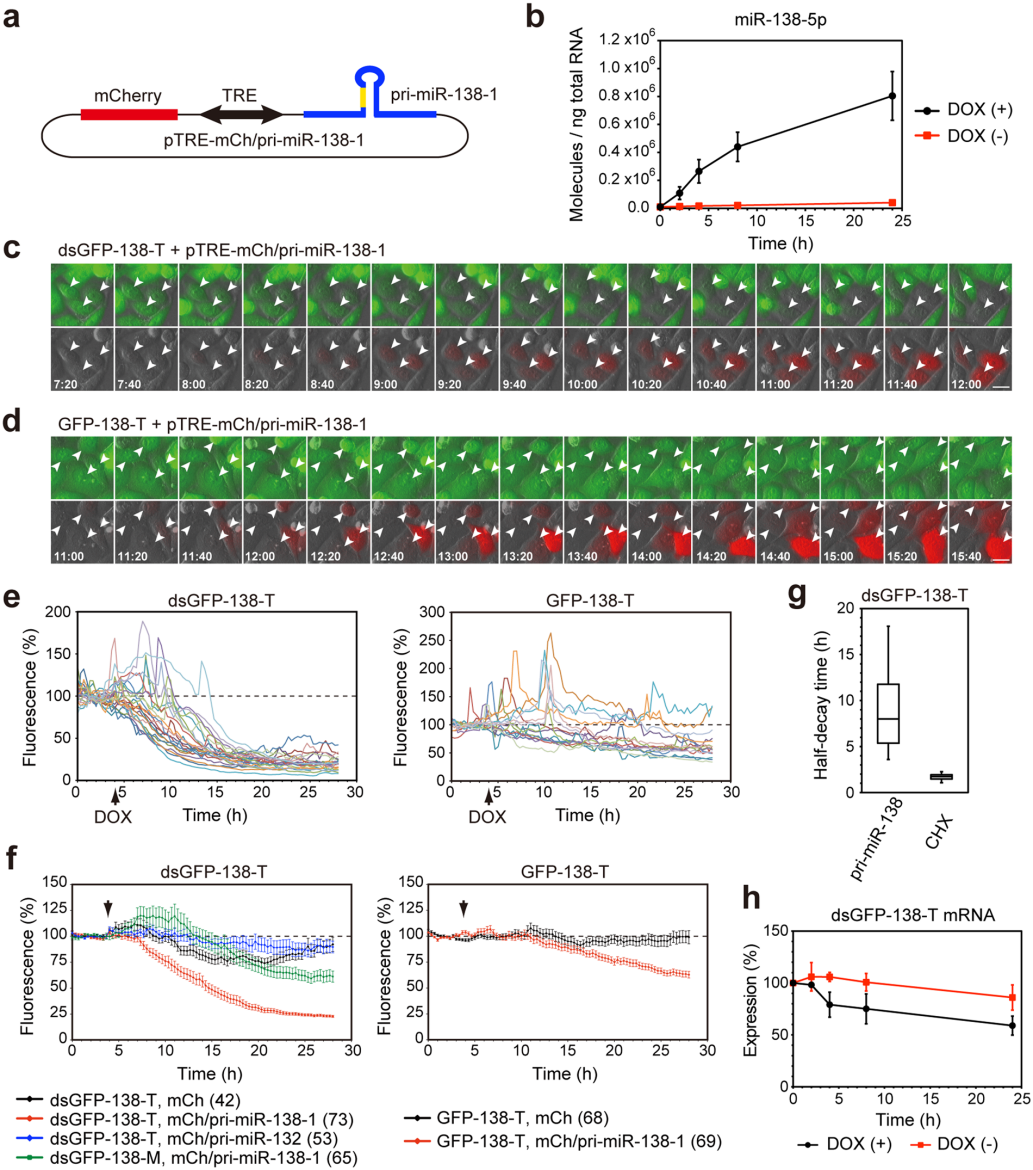
Time-lapse imaging of the miR-138-5p sensors in living cells. (**a**) pTRE-mCh/pri-miR-138-1 vector. (**b**) Stable HeLa cells expressing dsGFP-138-T were transfected with pTRE-mCh/pri-miR-138-1 and tTA, and the cells were incubated with or without 1 µg/mL doxycycline for 2, 4, 8 or 24 h. miR-138-5p expression was analyzed by quantitative RT-PCR. Data are expressed as mean ± SD (n = 3). (**c**,**d**) Stable HeLa cells expressing dsGFP-138-T (**c**) or GFP-138-T (**d**) were transfected with pTRE-mCh/pri-miR-138-1 and tTA, and expression of pri-miR-138-1 and mCherry were induced by doxycycline. Fluorescence of miR-138-5p sensors and mCherry were captured every 20 min at 37 °C. mCherry-expressing cells are indicated by arrowheads. Scale bars: 20 µm. (**e**) Representative time courses of fluorescence intensities of single cells expressing dsGFP-138-T (left) and GFP-138-T (right). Pri-miR-138-1 expression was induced at the indicated time (DOX). Note that sharp peaks were caused by cell-rounding during cell division. (**f**) Relative fluorescence intensities of dsGFP-138-T and dsGFP-138-M (left) and GFP-138-T (right) are presented as mean ± SEM. Numbers of cells analyzed are shown in parentheses. (**g**) Box-plot of half-decay time (T_1/2_) of dsGFP-138-T. The box represents the 25th and 75th percentiles. Whiskers show 5th and 95th percentiles. (**h**) Stable HeLa cells expressing dsGFP-138-T were transfected with pTRE-mCh/pri-miR-138-1 and tTA, and the cells were incubated with or without 1 µg/mL doxycycline for 2, 4, 8 or 24 h. The levels of dsGFP-138-T mRNA were analyzed by quantitative RT-PCR. Data are expressed as mean ± SD (n = 3).

Next, we tested whether dsGFP-based miRNA sensors can monitor the fast dynamics of miRNA activity in single living cells. We constructed the pTRE-mCherry/pri-miR-138-1 vector, which encodes 1) a 347-nucleotide (nt) genomic region containing a pri-miR-138-1 stem-loop and flanking regions with cis-acting elements that are required for miRNA processing^32,33^, 2) the gene for the fluorescent mCherry protein, under the control of 3) the bi-directional tetracycline-responsive element (TRE) promoter (Fig. 2a). We confirmed that the addition of doxycycline induced the expression of mature miR-138-5p in the cells that were transfected with the pTRE-mCherry/pri-miR-138-1 vector and the tetracycline-controlled transcriptional transactivator (tTA) expression vector (Fig. 2b). We then performed time-lapse imaging using stable HeLa cells expressing either dsGFP-138-T or GFP-138-T. The pTRE-mCherry/pri-miR-138-1 vector and the tTA expression vector were transfected into stable cells, and the fluorescence levels of either dsGFP-138-T or GFP-138-T were monitored every 20 min, at 37 °C. After pri-miR-138-1 expression and mCherry expression were induced via the addition of doxycycline, gradual decreases in dsGFP-138-T fluorescence were observed (Fig. 2c). Quantification of fluorescent intensity in individual cells showed the time course of dsGFP-138-T suppression (Fig. 2e,f). Reduction in dsGFP-138-T fluorescence was apparent approximately 4 h after pri-miR-138-1 induction (Fig. 2f), and median half-decay time (T_1/2_) of dsGFP-138-T was 8.0 h (n = 73) (Fig. 2g). For comparison, the T_1/2_ of dsGFP-138-T was measured in response to the protein translation inhibitor cycloheximide (CHX) and was found to be 1.7 h (n = 66) (Fig. 2g, Supplementary Fig. S2), which is consistent with the half-life of dsGFP^26^. We determined that neither the control pTRE-mCherry vector nor the pTRE-mCherry/pri-miR-132 vector suppressed dsGFP-138-T fluorescence (Fig. 2f). Furthermore, dsGFP-138-M, which has mutations within the seed region of the miR-138-5p binding sites, was also not effectively suppressed by pri-miR-138-1 (Fig. 2f). These results confirmed the specificity of the dsGFP-138-T sensor. Additionally, we observed a decrease in the mRNA levels of the dsGFP-138-T sensor, which was due to pri-miR-138-1 induction (Fig. 2h). In contrast to dsGFP-138-T, suppression of the GFP-138-T signal was slow, with a reduction of only approx. 40% occurring 24 h after pri-miR-138-1 induction (Fig. 2d–f), which is consistent with the results obtained from transfection with the miR-138-5p mimics (Fig. 1c,d,f). These results indicate that dsGFP-based miRNA sensors could be used to monitor the rapid dynamics of miRNA activity in single living cells.

### Effects of central and 3′ region mismatches on the decay kinetics of the miRNA sensor

**Figure 3:**
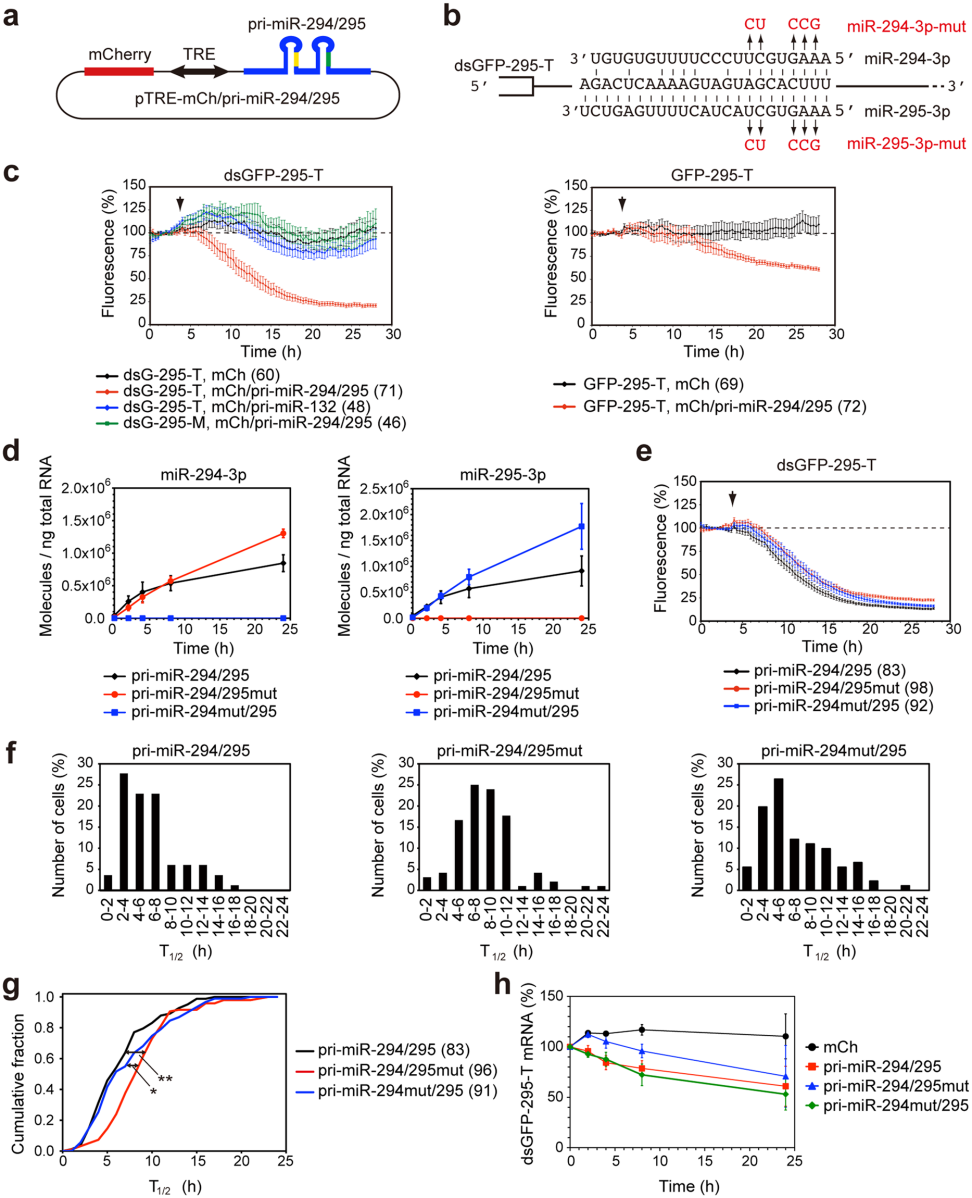
Time-lapse imaging of the miR-295-3p sensor in living cells. (**a**) pTRE-mCh/pri-miR-294/295 vector. (**b**) Sequences of miR-294-3p, miR-295-3p, and the miRNA target site in the dsGFP-295-T sensor. Mutations introduced into pTRE-mCh/pri-miR-294/295 are indicated by red. (**c**) Stable HeLa cells expressing dsGFP-295-T, dsGFP-295-M or GFP-295-T were transfected with pTRE-mCh/pri-miR-294/295 and tTA, and the expression of pri-miR-294/295 and mCherry were induced by doxycycline at the indicated time. Relative fluorescence intensities of dsGFP-295-T and dsGFP-295-M (left) and GFP-295-T (right) are presented as mean ± SEM. Numbers of cells analyzed are shown in parentheses. (**d**–**h**) dsGFP-295-T expressing cells were transfected with pTRE-mCh/pri-miR-294/295, pTRE-mCh/pri-miR-294/295mut, or pTRE-mCh/pri-miR-294mut/295 with tTA, and the expression of pri-miRNAs were induced by doxycycline. (**d**) Expression of miR-294-3p and miR-295-3p was analyzed by quantitative RT-PCR. Data are expressed as mean ± SD (n = 3). (**e**) Fluorescence of dsGFP-295-T was analyzed by time-lapse imaging. Relative fluorescence intensities of dsGFP-295-T are expressed as mean ± SEM. (**f**) Distribution of T_1/2_ of dsGFP-295-T in individual cells. (**g**) Cumulative distributions of T_1/2_. Numbers of cells analyzed are shown in parentheses. *P < 0.05, **P < 0.01 (Kruskal-Wallis test followed by Steel-Dwass test). (**h**) The levels of dsGFP-295-T mRNA were analyzed by quantitative RT-PCR. Data are expressed as mean ± SD (n = 3).

Next, we investigated whether mismatches outside of the seed region between a miRNA and the target sites in the miRNA sensor affect the decay kinetics of the miRNA sensor. miRNAs are often clustered with other miRNAs, and are co-expressed from a single polycistronic transcription unit^34^. Two mouse embryonic stem cell-specific miRNAs, miR-294-3p and miR-295-3p, are both encoded in the mir-290–295 cluster, and also have the same seed sequence^35,36^ (Fig. 3a,b). The target sequences of the dsGFP-295-T sensor are perfectly complementary to miR-295-3p, but not to miR-294-3p, to which they contain 7-nt mismatches in the central and 3′ regions (Fig. 3b). We constructed the pTRE-mCherry/pri-miR-294/295, which encodes a 469-nt genomic region containing the pri-miR-294 stem-loop, the pri-miR-295 stem-loop, and the flanking regions (Fig. 3a). The pTRE-mCherry/pri-miR-294/295 vector and the tTA expression vector were transfected into stable HeLa cells expressing dsGFP-295-T or GFP-295-T, and time-lapse imaging was performed as described above. The fluorescence of dsGFP-295-T was rapidly and specifically suppressed by induction of pri-miR-294/295, but not by induction of pri-miR-132, nor by induction of mCherry alone (Fig. 3c, Supplementary Fig. S1); whereas the decay rate of GFP-295-T was slow (Fig. 3c, Supplementary Fig. S1). As a control, we determined that dsGFP-295-M, which contains mutations within the seed region of the miR-294-3p/miR-295-3p binding sites, was not suppressed by pri-miR-294/295 (Fig. 3c).

To compare the decay kinetics of dsGFP-295-T when regulated by miR-294-3p vs. by miR-295-3p, we introduced mutations into the seed sequences of miR-295-3p and miR-294-3p in the pTRE-mCherry/pri-miR-294/295 vector (Fig. 3b). We confirmed that these mutations inhibited the expression of the corresponding miRNAs (Fig. 3d). The pTRE-mCherry/pri-miR-294/295 vector, or one of its mutants, was co-transfected with the tTA expression vector into cells expressing dsGFP-295-T, and the kinetics of the dsGFP-295-T suppression in single cells was analyzed using time-lapse imaging (Fig. 3e). The median T_1/2_ of dsGFP-295-T in individual cells expressing pri-miR-294/295, pri-miR-294/295mut, or pri-miR-294mut/295 was 5.4 h (n = 83), 8.0 h (n = 96), and 5.9 h (n = 91), respectively (Fig. 3f). Mutations of miR-295-3p, but not miR-294-3p, in the pri-miR-294/295 cluster significantly slowed the decay kinetics of the dsGFP-295-T sensor (Fig. 3g), suggesting that miR-295-3p in the pri-miR-294/295 cluster played a dominant role in the suppression of the dsGFP-295-T sensor. The slightly, but significantly, slower kinetics of miR-294-3p action compared to those of miR-295-3p (Fig. 3g) suggest that the mismatches in the central and 3′ regions affected the decay kinetics of dsGFP-based miRNA sensors. We analyzed the mRNA levels of the dsGFP-295-T sensor by quantitative RT-PCR. The reduction in the levels of dsGFP-295-T mRNA that was caused by miR-294-3p was slower than that caused by miR-295-3p (Fig. 3h). Because the mismatches outside of the seed sequence are reported not to affect the affinity between miRNA in complex with mouse Ago2 and target RNA^37^, the difference in kinetics may be attributable to the rate of target mRNA release and regeneration of the RISC. miR-295-3p, which is perfectly complementary to the dsGFP-295-T sensor, is assumed to cause endonucleolytic cleavage of mRNA in an Ago2-dependent manner^29–31^, whereas miR-294-3p, which contains mismatches to the target sites, is assumed to repress translation, and cause mRNA degradation without inducing mRNA cleavage because of the central mismatches^29,37–39^.

### Mathematical model of the miRNA-mediated regulation of the miRNA sensors

**Figure 4:**
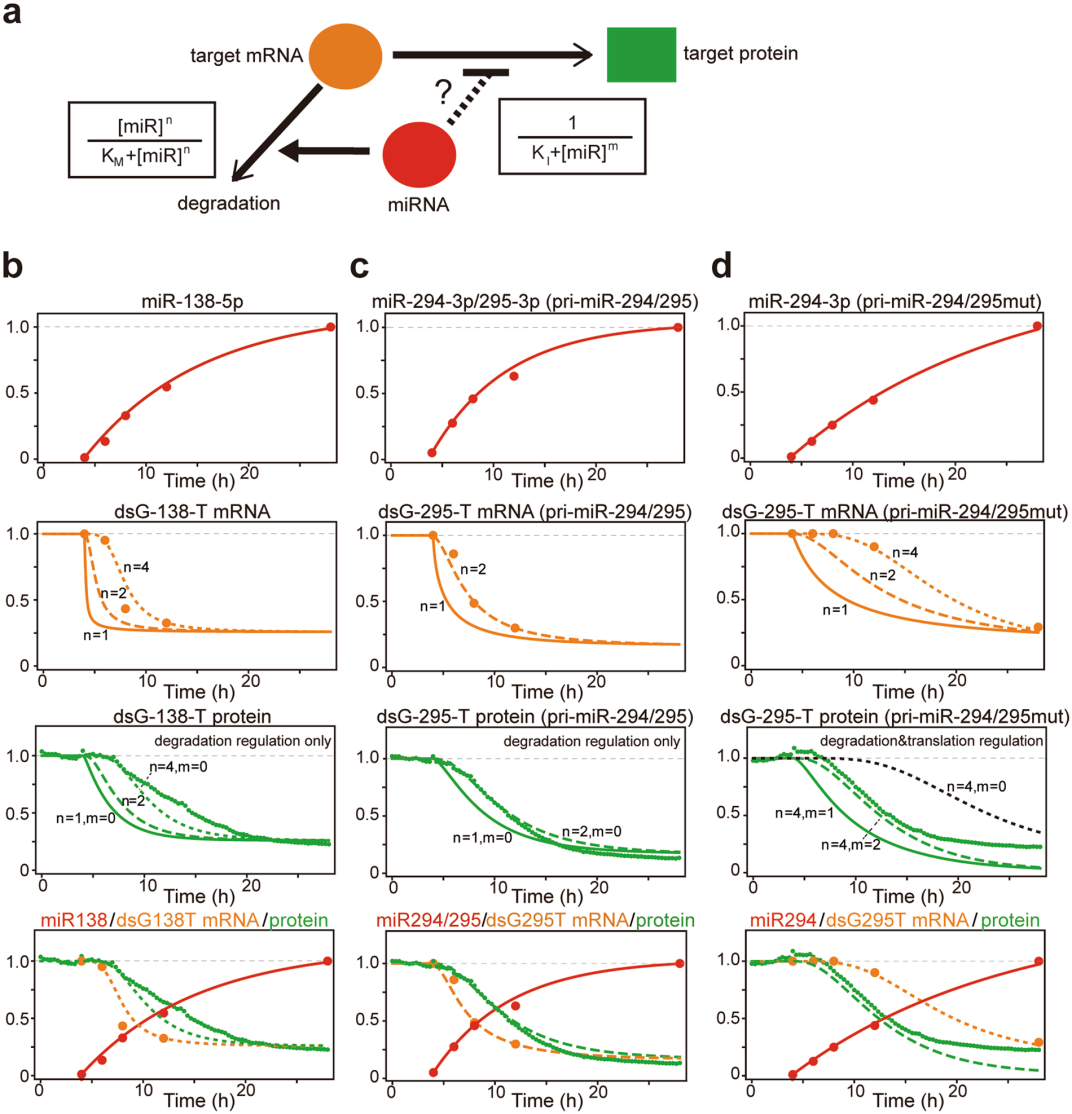
Mathematical model of the miRNA-mediated regulation of the miRNA sensors. (**a**) Models of miRNA function. The up-regulation of target mRNA degradation and the down-regulation of translation by miRNA are expressed by nonlinear functions of miRNA. The cooperativity in the regulations is expressed as *n* (up-regulation of degradation) and *m* (down-regulation of translation). (**b**–**d**) We attempted to reproduce the time series of the target protein (green) using the experimental data of the time series of the expression of the miRNA (red) and target mRNA (orange) as well as the measured half-lives of dsGFP-138-T and dsGFP-295-T. First, we obtained the degradation rate of the target protein from the measured half-lives (see text). Second, we searched for the parameter set for the dynamics of the miRNA and target mRNA, which reproduced the experimental data of the time series of the miRNA and target mRNA (red and orange dots, respectively). Using these parameters, which reproduced the data of miRNA and target mRNA, we estimated the time series of the target protein (green). (**b**) Decay of dsGFP-138-T by pri-miR-138-1 induction. Experimental data are derived from Fig. 2b,f and h. (**c**–**d**) Decay of dsGFP-295-T by pri-miR-294/295 induction (**c**) or pri-miR-294/295mut induction (**d**). Experimental data are derived from Fig. 3d,e and h.

To verify the aforementioned interpretation, we performed mathematical modeling using experimental data on the time courses of miRNA expression (Figs 2b, 3d), mRNA expression (Figs 2h, 3h) and the fluorescence of the miRNA sensors (Figs 2f, 3e). We considered the efficiency of the co-transfection of mCherry/pri-miRNAs and the tTA plasmids (Supplementary Fig. S3) to compensate for the mRNA expression data (Figs 2h, 3h). Because our results showed that the induction of miRNAs actually induced the decrease in the levels of the target mRNAs (Figs 2h, 3h), we presumed two possibilities— miRNAs regulate the expression of miRNA sensors through (1): the regulation of mRNA degradation only, and (2) both mRNA degradation and translational suppression (Fig. 4a). We attempted to identify the mechanisms that are more likely to underlie the observed decrease in the fluorescence of the miRNA sensors.

The observed decrease in the fluorescence of dsGFP-138-T and dsGFP-295-T under the induction of pri-miR-138-1 and pri-miR-294/295, respectively, could be explained by the mathematical model with the regulation of mRNA degradation only. By incorporating the measured half-life of the dsGFP-138-T protein into the model (1.7 h, Fig. 2g, Supplementary Fig. S2), we could estimate the time series of the dsGFP-138-T protein (Fig. 4b, the third panel from top) from the time series of the dsGFP-138-T mRNA (Fig. 4b, the second panel from top). The estimated time series of the dsGFP-138-T protein reproduced the observed delayed decrease in dsGFP-138-T fluorescence under pri-miR-138-1 induction (Fig. 4b, the third panel from top). We presumed that the delayed decrease (~4 h) in dsGFP-138-T fluorescence was due to the delayed decrease in its mRNA level. Similarly, for the experimental data sets of dsGFP-295-T under pri-miR-294/295 induction, the model with the regulation of mRNA degradation only, reproduced the time series of dsGFP-295-T fluorescence (Fig. 4c). Therefore, the mathematical model predicted that miRNAs should regulate the expression of the target mRNAs with perfectly complementary sites via mRNA degradation, probably through mRNA cleavage^29–31^, without translational repression.

In contrast, the mathematical model with the regulation of degradation only, could not reproduce the time series of the dsGFP-295-T fluorescence from the time series of the dsGFP-295-T mRNA under pri-miR-294/295mut induction. With the measured half-life of dsGFP-295-T (2.3 h, Supplementary Fig. S2), the model predicted that dsGFP-295-T fluorescence should have decreased with a delay of more than 8 h, which was inconsistent with the data (Fig. 4d, the third panel from top, black dashed line). When both mRNA degradation and translational suppression were assumed to occur, the model reproduced the observed time series of the dsGFP-295-T fluorescence under pri-miR-294/295mut induction (Fig. 4d, the third panel from top, green dashed line). Thus, our mathematical model predicted that the binding of miR-294-3p to the mismatch-containing target sites in the dsGFP-295-T mRNA should cause both translational repression and mRNA degradation, which is consistent with the mechanisms of miRNA-mediated gene regulation in animals^12^.

### Simultaneous imaging of miR-9-5p and miR-9-3p activation in single cells

**Figure 5:**
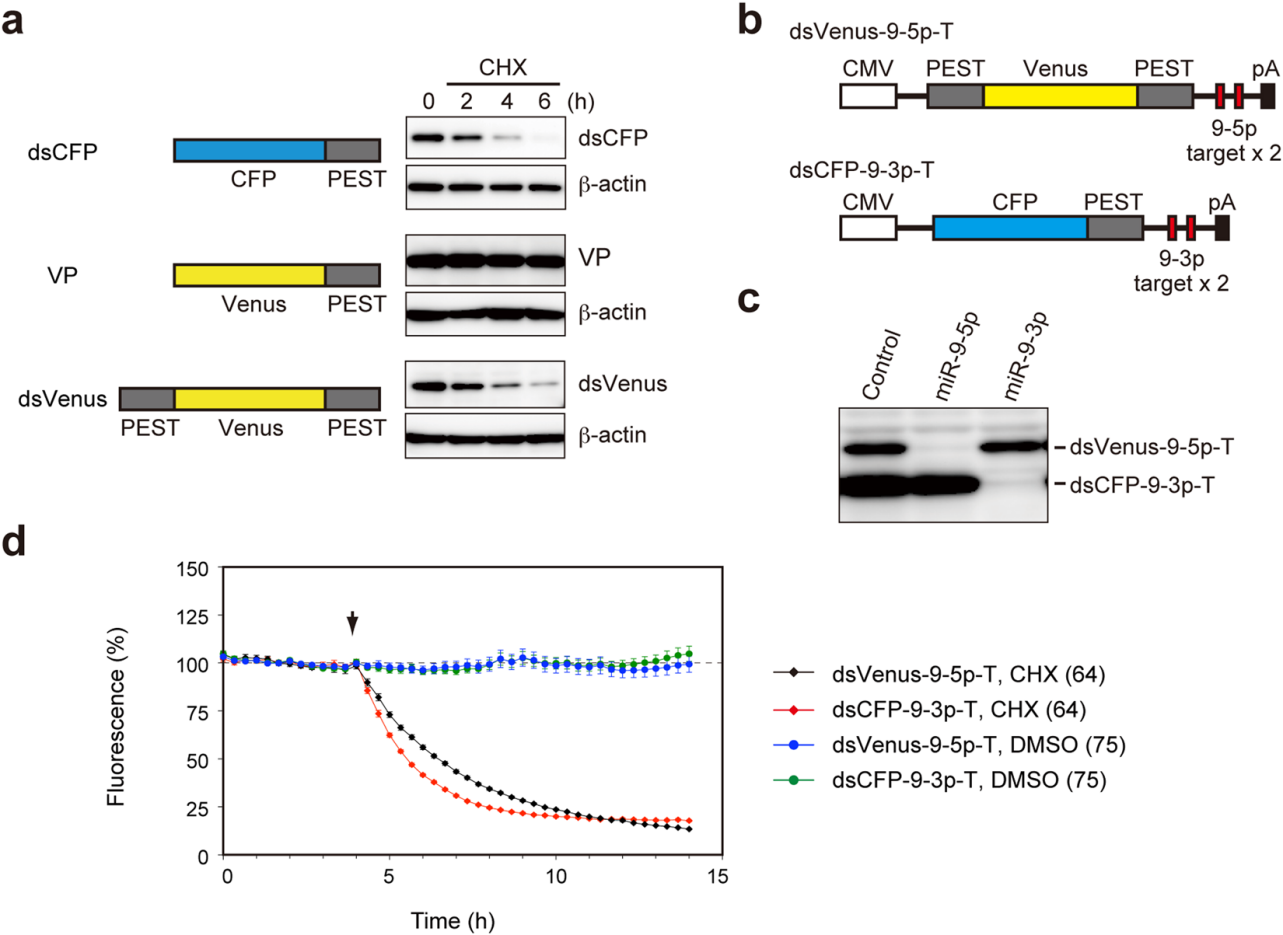
Development of miR-9-5p and miR-9-3p sensors using destabilized CFP and Venus. (**a**) HeLa cells transfected with dsCFP, VP, or dsVenus were treated with 100 µg/mL CHX and analyzed by western blotting with anti-GFP and anti-β-actin antibodies. (**b**) Structure of miR-9-5p and miR-9-3p sensors. (**c**) Stable HeLa cells expressing both dsVenus-9-5p-T and dsCFP-9-3p-T were transfected with 1 nM miR-9-5p, miR-9-3p, or control miRNA mimics and were analyzed by western blotting with anti-GFP antibody. (**d**) Stable HeLa cells expressing both dsVenus-9-5p-T and dsCFP-9-3p-T were treated with 100 µg/mL CHX or 0.1% DMSO. Fluorescence intensities of dsVenus-9-5p-T and dsCFP-9-3p-T were analyzed by time-lapse imaging and are presented as mean ± SEM. Numbers of cells analyzed are shown in parentheses.

**Figure 6:**
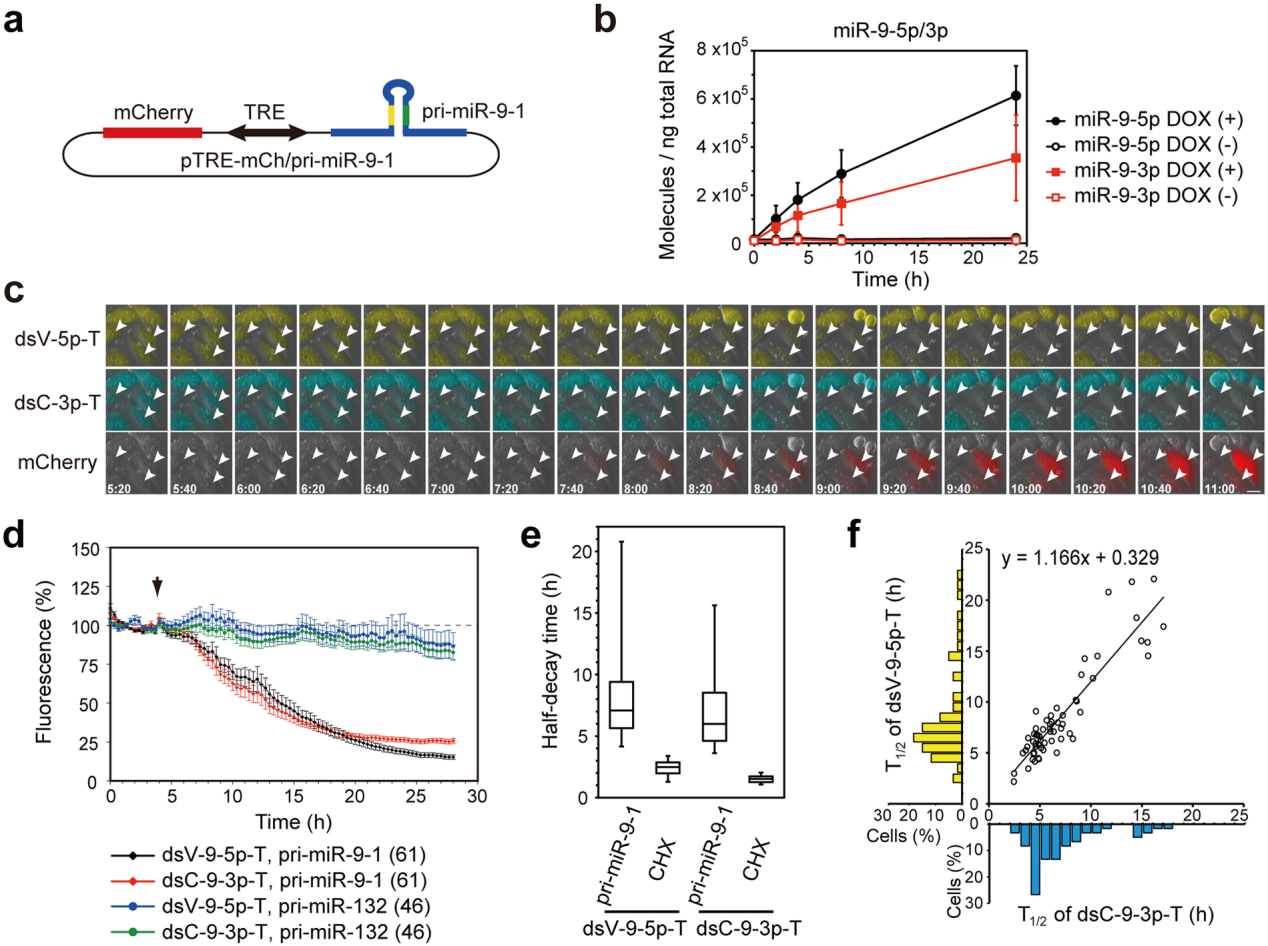
Dual-imaging of activation of miR-9-5p and miR-9-3p in living cells. (**a**) pTRE-mCh/pri-miR-9-1 vector. (**b**) Stable HeLa cells expressing both dsVenus-9-5p-T and dsCFP-9-3p-T were transfected with pTRE-mCh/pri-miR-9-1 and tTA, and the cells were incubated with or without 1 µg/mL doxycycline for 2, 4, 8 or 24 h. Expression of miR-9-5p and miR-9-3p were analyzed by quantitative RT-PCR. Data are expressed as mean ± SD (n = 5). (**c**) Stable HeLa cells expressing both dsVenus-9-5p-T and dsCFP-9-3p-T were transfected with pTRE-mCh/pri-miR-9-1 and tTA, and expression of pri-miR-9-1 and mCherry was induced by doxycycline. Fluorescence of dsVenus-9-5p-T, dsCFP-9-3p-T and mCherry were captured every 20 min at 37 °C. mCherry-expressing cells are indicated by arrowheads. Scale bar: 20 µm. (**d**) Relative fluorescence intensities of dsVenus-9-5p-T and dsCFP-9-3p-T are presented as mean ± SEM. Numbers of cells analyzed are shown in parentheses. (**e**) Box-plot of T_1/2_ of dsVenus-9-5p-T and dsCFP-9-3p-T. The box represents the 25th and 75th percentiles. Whiskers show 5th and 95th percentiles. (**f**) T_1/2_ of dsCFP-9-3p-T (x-axis) and dsVenus-9-5p-T (y-axis) in individual cells are plotted (60 cells). Marginal histograms show distributions of T_1/2_ of dsCFP-9-3p-T (bottom) and dsVenus-9-5p-T (left).

In the RISC loading process, one strand (the guide strand) of ~22-bp miRNA duplex is generally preferentially selected over the other strand (the passenger strand); however, in some cases, including the brain-specific miRNAs miR-9-5p and miR-9-3p, both strands are selected at similar levels^40,41^. It is unknown whether both miR-9-5p and miR-9-3p are activated concurrently at the single-cell level. To image the activation of miR-9-5p and miR-9-3p simultaneously in single cells, we developed a dual-imaging system that monitors the activities of the two miRNAs. First, we generated destabilized cyan fluorescent protein (dsCFP) and destabilized Venus protein (dsVenus). CFP was destabilized by addition of a PEST sequence at the C-terminus, as was the case with GFP (Fig. 5a). Venus was not destabilized by addition of a PEST sequence at the C-terminus, but the addition of a second PEST sequence at the N-terminus successfully destabilized Venus (Fig. 5a). We inserted two perfect-match complementary sequences against miR-9-5p or miR-9-3p into the 3′ UTR of dsVenus and of dsCFP, and termed these constructs dsVenus-9-5p-T and dsCFP-9-3p-T, respectively (Fig. 5b). We established stable HeLa cell lines expressing both dsVenus-9-5p-T and dsCFP-9-3p-T, and confirmed that dsVenus-9-5p-T and dsCFP-9-3p-T were specifically suppressed by miR-9-5p and miR-9-3p mimics, respectively (Fig. 5c). Time-lapse imaging of the stable HeLa cells treated with CHX showed that the median T_1/2_ values for dsVenus-9-5p-T and dsCFP-9-3p-T were 2.5 h and 1.5 h, respectively (n = 64) (Figs 5d, 6e), which are close to the half-life of dsGFP^26^.

Next, we analyzed the kinetics of miR-9-5p and miR-9-3p biogenesis and activation at the single-cell level. We constructed the pTRE-mCherry/pri-miR-9-1 vector, which encodes a 425-nt genomic region containing pri-miR-9-1 stem-loops and flanking regions (Fig. 6a). We confirmed that pTRE-mCherry/pri-miR-9-1 produced both miR-9-5p and miR-9-3p (Fig. 6b). We transfected the pTRE-mCherry/pri-miR-9-1 vector and the tTA expression vector into stable HeLa cells that express both dsVenus-9-5p-T and dsCFP-9-3p-T; we then performed dual imaging of dsVenus-9-5p-T and dsCFP-9-3p-T. After pri-miR-9-1 expression and mCherry expression were induced by the addition of doxycycline, the fluorescence levels of both dsVenus-9-5p-T and dsCFP-9-3p-T decreased with similar kinetics (Fig. 6c,d). Induction of pri-miR-132 did not alter the fluorescence of dsVenus-9-5p-T or dsCFP-9-3p-T (Fig. 6d), confirming specificity of the effect elicited by inducing pri-miR-9-1. The median T_1/2_ values for dsVenus-9-5p-T and dsCFP-9-3p-T were 7.0 h and 6.0 h, respectively (n = 60) (Fig. 6e). Slightly slower decay of dsVenus-9-5p-T than that of dsCFP-9-3p-T may be due to the longer half-life of dsVenus than that of dsCFP (Figs 5d, 6e). We then analyzed correlations of T_1/2_ values for dsVenus-9-5p-T and dsCFP-9-3p-T at the single-cell level. The T_1/2_ for dsVenus-9-5p-T was positively correlated with that for dsCFP-9-3p-T (Spearman’s correlation coefficient, rs = 0.84, P < 10^−9^, n = 60) (Fig. 6f). As a comparison, we performed the same experiments using a stable HeLa cell line expressing dsVenus-9-5p-T and dsCFP-9-5p-T. The correlations of T_1/2_ values for dsVenus-9-5p-T and dsCFP-9-3p-T were as strong as those of dsVenus-9-5p-T and dsCFP-9-5p-T (rs = 0.76, P < 10^−7^, n = 55) (Supplementary Fig. S4). These results suggest that two functional miRNAs, miR-9-5p and miR-9-3p, were produced from a single hairpin precursor in single cells, and that miR-9-5p and miR-9-3p were activated concurrently and with similar kinetics.

### Comparison of the kinetics of miRNA activation

**Figure 7:**
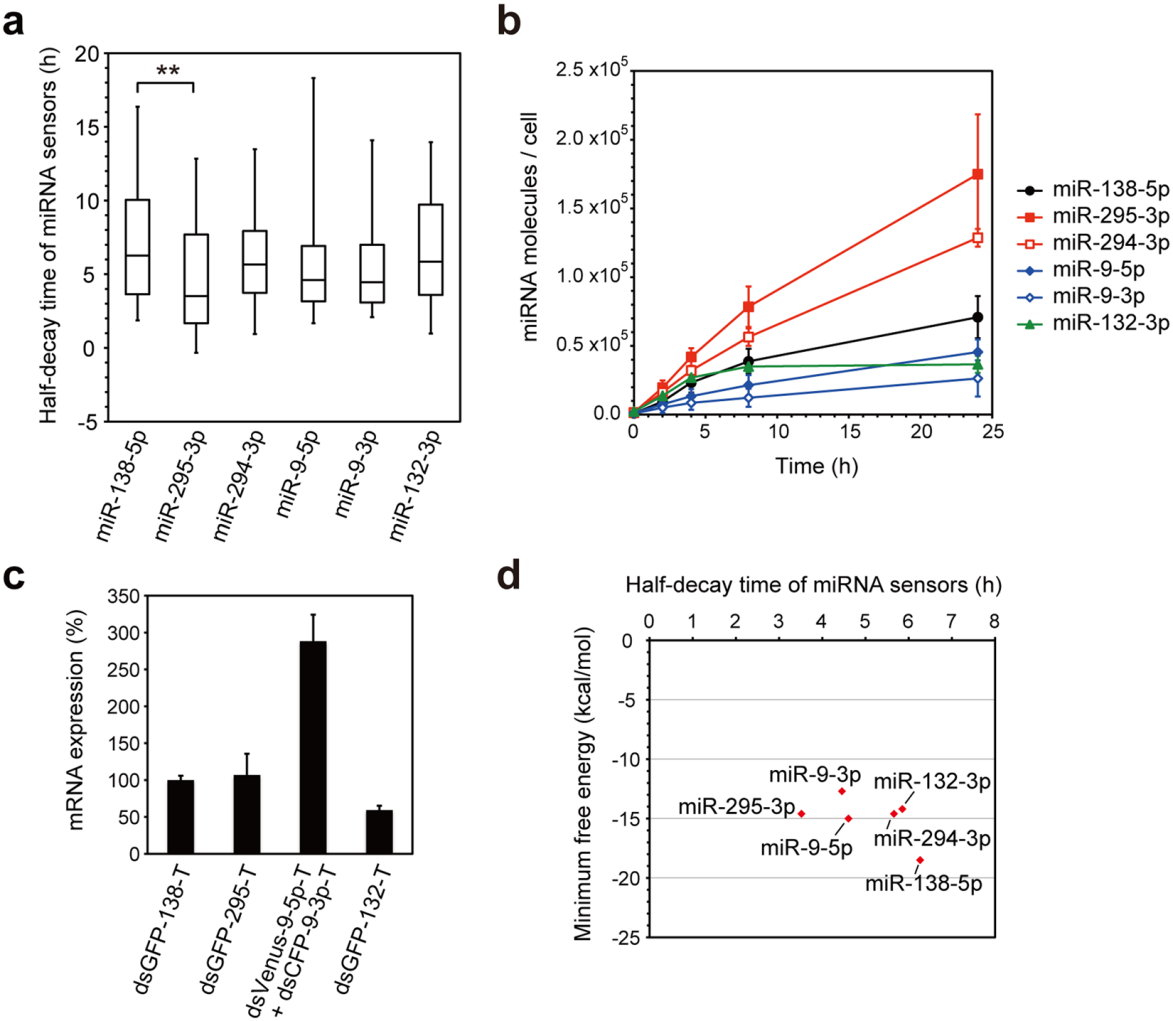
Comparison of the kinetics of miRNA activation. (**a**) Box-plot of the half-decay time of the dsFP-based miRNA sensors. Median half-decay time periods of the corresponding dsFP-based miRNA sensors caused by CHX (Fig. 5d, Supplementary Fig. S2, S5) were subtracted from the half-decay time periods of dsGFP-138-T, dsGFP-295-T, dsVenus-9-5p-T and dsCFP-9-3p-T, and dsGFP-132-T, whose decay was caused by the induction of pri-miR-138-1 (Fig. 2f), pri-miR-294mut/295 or pri-miR-294/295mut (Fig. 3e), pri-miR-9-1 (Fig. 6d), and pri-miR-132 (Supplementary Fig. S5), respectively. The box represents the 25th and 75th percentiles. Whiskers show 5th and 95th percentiles. **P < 0.01 (Kruskal-Wallis test followed by Steel-Dwass test). (**b**) Molecule numbers of the mature miRNAs in a single transfected cell were calculated from the miRNA expression data (Figs 2b, 3d, 6b, Supplementary Fig. S5), the total amount of RNA in a single HeLa cell (32.5 pg/cell), and the co-transfection efficiency of mCherry/pri-miRNAs and tTA (Supplementary Fig. S3). Data are expressed as mean ± SD (n = 3–5). (**c**) Relative expression levels of the miRNA sensor mRNAs in the stable HeLa cell lines were analyzed by quantitative RT-PCR. The total of dsVenus-9-5p-T and dsCFP-9-3p-T mRNAs is shown, because it was technically difficult to discriminate between them because of their sequence similarity. Data are expressed as mean ± SD (n = 3). (**d**) The minimum free energies of the binding of the 8 nucleotides at the 5′-terminal of the miRNAs to the target sites of the miRNA sensors were predicted by RNAhybrid^42^.

We investigated the activation kinetics of miR-138-5p, miR-295-3p, miR-294-3p, miR-9-5p, and miR-9-3p in single living cells. In a similar way, we analyzed the time course of the activation of a neuron-enriched miRNA, miR-132-3p, using the dsGFP-132-T sensor (Supplementary Fig. S5). We compared the timescales of the activation of these miRNAs by subtracting the median half-decay time of the dsFP-based miRNA sensors caused by CHX (Fig. 5d, Supplementary Fig. S2, S5) from the half-decay time of the dsFP-based miRNA sensors caused by pri-miRNA induction (Figs 2f, 3e, 6d, Supplementary Fig. S5). The median half-decay time of the dsGFP-295-T sensor caused by miR-295-3p induction (3.5 h) was the shortest, with its half-decay being significantly faster than that of dsGFP-138-T caused by miR-138-5p induction (6.3 h) (Fig. 7a). To reveal the underlying mechanisms of the relatively rapid kinetics of miR-295-3p activation, we compared the miRNA expression levels, mRNA levels of the miRNA sensors, and binding energies of the miRNAs and target mRNAs. We calculated the molecule number of mature miRNAs in a single transfected cell by using the data of the quantitative RT-PCR analysis of mature miRNAs (Figs 2b, 3d, 6b, Supplementary Fig. S5), total amount of RNA in a single HeLa cell (32.5 +/− 4.0 pg; n = 3), and transfection efficiency of pri-miRNAs (Supplementary Fig. S3). The rate of miR-295-3p biogenesis was higher than those of the biogenesis of other miRNAs (Fig. 7b). Meanwhile, the mRNA level of dsGFP-295-T was similar to that of dsGFP-138-T (Fig. 7c). The free energies of the binding of the 8 nucleotides at the 5′-terminal of miR-295-3p to the target sites of the miRNA sensor, which was predicted by RNAhybrid^42^, was similar to those of the other miRNAs (Fig. 7d). These results suggest that the relatively higher rate of miR-295-3p biogenesis accounts for the rapid kinetics of miR-295-3p activation.

### Kinetics of miR-132-3p activation in living neurons

**Figure 8:**
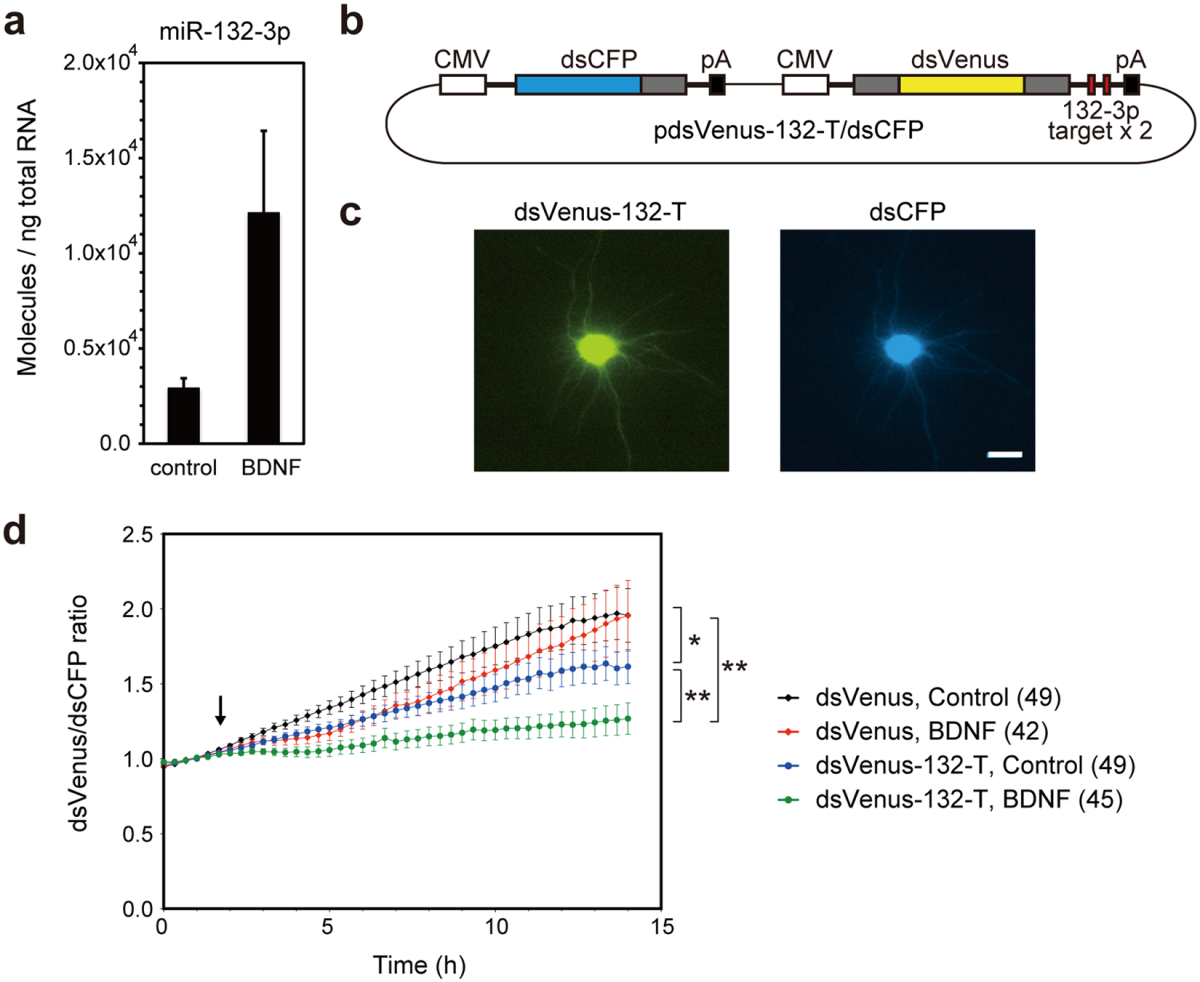
Time-lapse imaging of miR-132-3p activation in living neurons. (**a**) Cortical neurons (DIV 5) were stimulated with 50 ng/mL BDNF for 12 h. Expression of miR-132-3p was analyzed by quantitative RT-PCR. Data are expressed as mean ± SD (n = 6). (**b**) pdsVenus-132-T/dsCFP vector. (**c**) Cortical neurons were transfected with pdsVenus-132-T/dsCFP. Fluorescence of dsVenus-132-T (left) and dsCFP (right) are shown. Scale bars: 20 µm. (**d**) Neurons were transfected with pdsVenus/dsCFP or pdsVenus-132-T/dsCFP and were analyzed by time-lapse imaging. Fluorescence of dsVenus and dsCFP were captured every 20 min at 37 °C. BDNF (50 ng/mL) was added at the indicated time. The dsVenus/dsCFP ratio are presented as mean ± SEM. Numbers of cells analyzed are shown in parentheses. *P < 0.05, **P < 0.01 (two-way repeated measures ANOVA).

Next, we investigated the kinetics of biogenesis and activation of an endogenous miRNA under physiological conditions. miR-132-3p is a neuron-enriched miRNA that regulates neuronal development and function^43,44^. The neurotrophin BDNF increases the expression of miR-132-3p in cortical neurons by stimulating the transcription of pri-miR-132 in a CREB-dependent manner^44,45^. We confirmed that the expression of miR-132-3p in rat cortical neurons was increased upon BDNF stimulation (50 ng/mL, 12 h) (Fig. 8a). To monitor the activation kinetics of miR-132-3p in living neurons, we generated a miR-132-3p sensor, dsVenus-132-T, by inserting two perfect-match complementary sequences of miR-132-3p into the 3′ UTR of dsVenus. For normalization, we inserted the dsCFP cassette under the control of the CMV promoter in the plasmid encoding dsVenus-132-T. The resultant plasmid, pdsVenus-132-T/dsCFP, produced two independent transcripts, dsVenus-132-T and dsCFP, driven by each CMV promoter (Fig. 8b). pdsVenus-132-T/dsCFP was transfected into primary rat neurons and the fluorescence levels of dsVenus-132-T and dsCFP were monitored every 20 min at 37 °C (Fig. 8c,d). The baseline dsVenus-132-T/dsCFP ratio increased slightly with time (Fig. 8d), probably because the half-life of dsVenus is slightly longer than that of dsCFP (Figs 5d, 6e). Soon after the addition of BDNF, the dsVenus-132-T/dsCFP ratio was significantly reduced compared to that of the control (Fig. 8d). The decrease in the dsVenus-132-T/dsCFP ratio was apparent 2-3 h after BDNF addition, suggesting that biogenesis of miR-132-3p, from transcription of pri-miR-132 to activation of mature miR-132-3p, occurred on this timescale. The ratio of the control dsVenus without miR-132-3p target sites to dsCFP was not affected by BDNF stimulation (Fig. 8d). These results indicate that dsFP-based miRNA sensors could be used for monitoring the fast kinetics of endogenous miRNA activation under physiological conditions.

## Discussion

We have developed a novel method for real-time imaging of the rapid dynamics of miRNA activity using dsFPs, and we have revealed the kinetics of miRNA biogenesis and activation in living HeLa cells and neurons. Compared to stable fluorescent proteins, dsFPs are suitable for imaging of the relatively fast changes that occur in miRNA activity because of the short half-lives of dsFPs—their half-lives are even shorter than those of luciferase reporter proteins (firefly luciferase = 3 h, Renilla luciferase = 5.3 h)^46,47^. The dsFP-based method has advantages over luciferase-based bioluminescent imaging methods in terms of signal intensity and non-necessity of substrate addition. Furthermore, the dsFP-based method directly monitors miRNA activity, rather than the expression of mature miRNA that molecular beacons detect. Our time-lapse imaging experiments demonstrated that dsFP-based miRNA sensors detected onset of miRNA activation as early as approx. 2–4 h after pri-miRNA induction (Figs 2f, 3e, 6d, 8d, Supplementary Fig. S5), indicating that dsFP-based miRNA sensors allow for analyzing the fast kinetics of miRNA action, without the need to collect samples at multiple time points. Real-time imaging of miRNA activity using dsFPs will provide insight into the rapid temporal dynamics of miRNA activity in living cells.

The time-lapse imaging showed that the half-decay time of the dsFP-based miRNA sensors, whose decay was caused by induction of pri-miRNAs, ranged from 3.5 h (miR-295-3p) to 6.3 h (miR-138-5p) (Fig. 7a). The kinetics of miRNA activation was likely to have been affected by the rates of mature miRNA biogenesis. The difference in the rates of miRNA biogenesis (Fig. 7b) might be partly explained by the difference in the secondary structures of the pri-miRNAs or pre-miRNAs, which affect the cleavage efficiency of the Drosha and Dicer proteins^48–50^. However, the miRNA expression levels did not necessarily correlate with the kinetics of their activation. For example, the initial rates (up to 8 h) of the biogenesis of miR-138-5p and miR-132-3p were higher than those of miR-9-5p and miR-9-3p (Fig. 7b), whereas the kinetics of the activation of miR-138-5p and miR-132-3p were slower than those of miR-9-5p and miR-9-3p (Fig. 7a). The inconsistency in the expression and activity of miRNA has also been reported previously^17^. This discrepancy might be explained by the relatively higher expression levels of the dsVenus-9-5p-T/dsCFP-9-3p-T mRNAs (Fig. 7c, shown in total), which may have increased the frequency of interactions with miR-9-5p/3p. Alternatively, it might be caused by difference in the efficiency of the Ago loading process^51^, and/or the presence of Ago-unloaded, nonfunctional miRNA molecules.

miRNAs regulate the levels of target proteins by two mechanisms; translational repression and mRNA degradation^12^. We constructed mathematical models to determine the mechanisms that were more likely to explain the observed decrease in the fluorescence of the miRNA sensors. The model predicted that miRNAs should regulate target mRNAs with perfectly complementary sites via mRNA degradation without translational repression, whereas they should regulate the mRNAs with mismatch-containing complementary sites via both translational repression and mRNA degradation. To reproduce the time series of the fluorescence of the miRNA sensors, cooperativity of miRNA function in both the regulation of mRNA degradation (i.e. *n* = 2 or 4, Fig. 4b–d) and translational repression (i.e. *m* = 2, Fig. 4d) was required. Although the molecular mechanism of the cooperatively is unclear, it is possible that the cooperative action of two miRNA-binding sites in the miRNA sensors might be partly involved in the observed cooperativity^28,52^. In our mathematical models, time points for the miRNA and mRNA quantification were small compared to those for the protein levels, due to the necessity to lyse cells. Further studies, such as imaging of miRNA and mRNA levels using molecular beacon techniques^13–16,53^ in conjunction with the imaging of the miRNA activity, might potentially improve the relevance of the mathematical modeling.

We have established the dual-imaging system that simultaneously monitors activities of two different miRNAs using dsVenus and dsCFP. Using this system, we succeeded in imaging concurrent activation of miR-9-5p and miR-9-3p, which are both generated from pre-miR-9-1, at the single-cell level. Our results demonstrated that two distinct functional miRNAs could be generated from single pre-miRNA in single cells. The miR-9-5p/3p miRNAs are highly expressed in the developing and adult brain, and they play critical roles in the regulation of neural development and of brain functions^41,54,55^. miR-9-5p and miR-9-3p might regulate neural differentiation in a cooperative manner. Based on experimental data using transfection of miRNA mimics and mathematical modeling, RISC loading of guide-strand miRNA is proposed to be a slow rate-limiting process in miRNA activation, probably due to limited intracellular levels of Ago proteins^51,56,57^. In our study, the activation kinetics of two miRNAs (miR-9-5p and miR-9-3p) were positively correlated, and their timescales were similar to those of single miRNAs (miR-138-5p, miR-295-3p and miR-132-3p), suggesting that the RISC loading step is not rate limiting in HeLa cells. This difference may be explained by difference of cell types or differences of methodology: transfection of miRNA mimics versus induction of pri-miRNAs.

One critical product of the regulatory effects of miRNA is the down-regulation of miRNA target proteins. By comparing the decay kinetics of dsGFP-based and GFP-based miRNA sensors, we have demonstrated that the stability of the target proteins greatly affects the output of miRNA activity, which is suggested in mathematical simulations^51^. Proteomic analyses using stable isotope labeling with amino acids in cell culture (SILAC) have determined the half-lives of more than 4,000 proteins in mammalian cells; these range from less than one hour to several hundreds of hours, with different studies yielding median values of 36 h^58^ and 46 h^59^. Whichever value is more accurate, the median half-life for this large number of proteins is much longer than the timescale of miRNA biogenesis and activation estimated in this study; this indicates that the stability of miRNA target proteins, rather than miRNA biogenesis, is the dominant rate-limiting factor in miRNA-mediated gene silencing. For example, even if miRNA is activated within a time frame of several hours to repress the synthesis of a target protein with a half-life of the median value (36 h), it takes 3 days for the target protein levels to decrease to 25% in non-dividing cells. The slow output rate (the rate of decrease of target protein levels), in contrast to the fast input rate (the rate of miRNA activation), may be adequate for slow physiological processes, including differentiation and development; but this seems unsuitable for the regulation of rapid processes such as circadian activities. This apparent inconsistency in kinetics can be rationalized by several biological aspects of miRNA action. First, many miRNAs act as fine-tuning regulators rather than as binary on/off-switches^1^. Thus, subtle changes of target protein levels are physiologically relevant. In fact, large-scale proteomic analyses have shown that miRNAs have only modest effects on target protein levels^38,39^. Second, a bioinformatic analysis predicts that miRNAs tend to target relatively short half-life proteins^51^. Third, protein concentrations are decreased by a combination of degradation and cell-growth-mediated dilution^60^. Thus, miRNA target proteins can be efficiently removed in actively dividing cells such as stem cells^61^. Fourth, miRNAs can suppress target protein expression in a preventive manner. In our study, GFP-based miRNA sensors were efficiently suppressed when they were transfected with corresponding miRNAs at the same time (Fig. 1b, Supplementary Fig. 1), but they were not effectively suppressed when miRNAs were transfected or induced afterward (Figs 1c,d,f, 2d–f, 3c, Supplementary Fig. S1). Thus, miRNAs can effectively suppress target protein levels if miRNAs already exist before the induction of target protein synthesis. This preventive role of miRNAs may be beneficial when miRNAs need to suppress long half-life proteins in slowly dividing or non-dividing, differentiated cells. Furthermore, the fast kinetics of miRNA biogenesis and activation may be physiologically critical in the regulation of transitions from one cellular status to another, where dynamic changes in transcriptomes occur. In such situations, miRNAs are assumed to be induced concurrently with numerous mRNAs, including collaterally transcribed adverse mRNAs that need to be suppressed by miRNAs.

In summary, we have established a method for the real-time imaging of miRNA activity in single living cells, and we have estimated the time period needed to complete miRNA biogenesis and activation. Our results suggest that the stability of miRNA target proteins is the rate-limiting factor of miRNA-mediated gene silencing, and we infer a preventive mechanism of miRNA function. miRNA sensors using high-turnover fluorescent proteins are expected to provide insight into the spatiotemporal dynamics of miRNA activity *in vivo*, and are also expected to be useful in the screening of chemicals or proteins that regulate miRNA biogenesis.

## Methods

### Plasmids

The destabilized GFP expression vector (pdsGFP)^26^ was constructed by inserting the PEST sequence of mouse ornithine decarboxylase (amino acids 422–461), with a stop codon, into the BglII and HindIII sites of pEGFP-C1 (Clontech). The GFP expression vector with a stop codon (pGFP-stop) was constructed by inserting oligonucleotides containing a stop codon into the BspEI and BglII sites of pEGFP-C1. The destabilized CFP expression vector (pdsCFP) was constructed by replacing the EGFP cassette of pdsGFP with the ECFP sequence of pECFP-1 (Clontech) using the NheI and BspE1 sites. For the construction of the destabilized Venus expression vector (pdsVenus), the EGFP cassette of pdsGFP was replaced with Venus^62^ using the NheI and BspE1 sites, and the Venus-PEST cassette was cloned into NheI/BamHI sites of pcDNA3.1/Hygro (Invitrogen); the second PEST sequence then was inserted into the 5′ terminus of Venus-PEST using the NheI site. Double stranded oligonucleotides containing two copies of complementary sequences to miR-138-5p, miR-295-3p, miR-9-5p, miR-9-3p, or miR-132-3p were cloned into the SalI and BamHI sites in the 3′ UTR of pdsGFP, pGFP-stop, or pdsCFP, or were cloned into the BamHI and XhoI sites in the 3′ UTR of pdsVenus. As controls, mutations were introduced in the seed regions of the miRNA binding sites. For the construction of plasmids encoding both dsVenus and dsCFP, a dsCFP cassette, with a CMV promoter and polyA signal, was inserted into the NruI site of pdsVenus with or without miR-132-3p target sequences. For the construction of tetracycline-responsive vectors expressing both pri-miRNA and mCherry^63^, cDNA encoding mCherry was first cloned into multiple cloning site II of pTRE-tight-BI (Clontech), which we then termed pTRE-mCherry. Then, the approx. 350–500 base genomic region of mouse pri-miR-138-1, pri-miR-294/295, pri-miR-132, or pri-miR-9-1 were PCR-amplified and cloned into multiple cloning site I of pTRE-mCherry. Site-directed mutagenesis of pri-miR-294/295 was performed using the QuikChange II Site-Directed Mutagenesis Kit (Stratagene). The tetracycline-controlled transcriptional transactivator expression vector, pTet-ON-Advanced, was obtained from Clontech. Primers and oligonucleotides used in this study are shown in Supplementary Table S1.

### Cell culture

HeLa cells were maintained in Dulbecco’s modified Eagle’s medium (DMEM) (Nacalai Tesque) supplemented with 10% fetal bovine serum (FBS) (Equitech-Bio), 50 units/mL penicillin, and 50 µg/mL streptomycin (Nacalai Tesque) in a 5% CO_2_ incubator at 37 °C. To generate stable cell lines expressing GFP, dsGFP, or dsCFP-based miRNA sensors, plasmids encoding GFP/dsGFP/dsCFP-miRNA-target were linearized by ApaLI digestion, and were transfected into HeLa cells. Cells were cultured in 96-well plates at low density in the presence of 400 µg/mL G418 (Nacalai Tesque), and single colonies were isolated. Stable cell lines were maintained in medium containing 100 µg/mL G418. To generate stable cell lines expressing dsVenus-based miRNA sensors, plasmids encoding pcDNA3.1/Hygro-dsVenus-miRNA-target were linearized by FspI digestion and were transfected into HeLa cells. Single colonies were screened by culturing cells in the presence of 200 µg/mL hygromycin B (Wako). Stable cell lines were maintained in medium containing 50 µg/mL hygromycin B. For maintenance of stable cell lines used for time-lapse imaging, 10% Tet System Approved FBS (Clontech) was used instead of normal FBS. Primary cultures of cortical neurons were prepared as described previously^64^, with modifications. Briefly, the neocortex was excised from postnatal day 1 Wistar rats and digested at 37 °C for 45 min with papain (Worthington) in dissociation medium (81.8 mM Na_2_SO_4_, 30 mM K_2_SO_4_, 12 mM MgCl_2_, 0.25 mM CaCl_2_, 8 mM HEPES, 20 mM glucose, and 0.001% phenol red, pH 7.5). After rinsing with Opti-MEM (Life Technologies) and trituration, the cells were plated on polyethyleneimine-coated dishes at a density of 1 × 10^5^ cells/cm^2^. The cells were maintained in Neurobasal A medium (Life Technologies) supplemented with 2% B27 (Life Technologies), 0.32 mM L-glutamine, 50 units/mL penicillin, 50 µg/mL streptomycin, and 25% Neuron Medium (Sumitomo Bakelite) in a 5% CO_2_ incubator at 37 °C. All animal studies were carried out in accordance with the guidelines and approval from the Animal Experiments Committee at the RIKEN.

### Transfection

Transfection of plasmids into HeLa cells was performed using the TransIT-LT1 reagent (Mirus) according to the manufacturer’s instructions. Transfection of miRNA mimics into HeLa cells and transfection of plasmids into neurons were performed using LipofectAMINE 2000 (Life Technologies) according to the manufacturer’s instructions. Synthetic miRNA mimics were obtained from Sigma (hsa-miR-138-5p, hsa-miR-132-3p, hsa-miR-9-5p, hsa-miR-9-3p, and negative control RNA) or Ambion (mmu-miR-295-3p).

### Western blotting

Transfected cells were washed once with PBS and lysed in SDS-PAGE sample buffer. To analyze turnover of dsCFP and dsVenus, the cells were treated with 100 µg/mL cycloheximide (CHX) for varying times before cell lysis. Cell lysates were separated by SDS-PAGE and transferred onto polyvinylidene fluoride membrane (Millipore) by electroblotting. After blocking with 5% skim milk in PBS containing 0.1% Tween 20 (PBS-T) for 1 h at room temperature, membranes were immunoblotted with mouse anti-GFP (Medical & Biological Laboratories or Santa Cruz) or anti-β actin (Sigma) antibody for 1 h at room temperature. After washing three times with PBS-T, the membrane was incubated with anti-mouse IgG conjugated with horseradish peroxidase (GE Healthcare) for 1 h at room temperature. After washing three times with PBS-T, immunoreactive bands were visualized with the ECL detection system (GE Healthcare), and were captured using a luminescent image analyzer (LAS-4000 mini, GE Healthcare).

### Confocal microscopy

Transfected cells grown on glass coverslips were washed once with PBS and fixed in 4% formaldehyde in PBS for 15 min. After washing three times with PBS, the coverslips were mounted with Vectashield with DAPI (Vector Laboratories) and observed under a confocal fluorescence microscope (FV1000, Olympus) with a 20× objective.

### Flow cytometry

Transfected cells were collected, washed once with PBS, and fixed with 2% paraformaldehyde in PBS on ice. GFP fluorescence was analyzed by flow cytometry (Becton Dickinson, LSR) at the Support Unit for Bio-Material Analysis in the RIKEN BSI Research Resources Center (RRC). Data were analyzed using FlowJo software (TreeStar).

### Quantitative RT-PCR

Total RNA was purified using Trizol reagent (Life technologies) according to the manufacturer’s instructions. cDNA was synthesized by reverse transcription using ReverTra Ace qPCR RT master mix (Toyobo) according to the manufacturer’s instructions. mRNA expression levels of miRNA sensors were analyzed by real-time PCR using Thunderbird SYBR qPCR mix (Toyobo). GFP primers were used to detect miRNA sensors, and GAPDH primers were used to normalize miRNA sensor expression. PCR reactions were run using the Applied Biosystems 7900HT as follows: 95 °C for 1 min, followed by 40 cycles of 95 °C for 15 sec and 60 °C for 45 sec. Real-time RT-PCR for mature miRNAs were performed by using TaqMan microRNA assay (Applied Biosystems) according to the manufacturer’s protocol. Ten nanograms of total RNA was used for reverse transcription. Synthetic miRNA oligoribonucleotides that were used for obtaining the standard curves of the absolute quantification of miRNAs were purchased from FASMAC Co. Ltd. The sequences of the real-time PCR primers and synthetic miRNAs are described in the Supplementary Table S1.

### Time-lapse imaging

Stable HeLa cell lines expressing miRNA sensors plated in 35-mm glass bottom dishes (MatTek) were transfected with pTet-ON-Advanced and pTRE-mCherry-pri-miRNA vectors. The next day, cells were washed once with PBS and cultured in DMEM without phenol red (Nacalai Tesque) supplemented with 10% Tet System Approved FBS and 4 mM glutamine. The cells were then processed for time-lapse imaging using an incubator fluorescence microscope system (Olympus, LCV110) equipped with a 40× objective lens. Cells were maintained at 37 °C with 5% CO_2_ during experiments. Fluorescent and differential interference contrast (DIC) images were obtained every 20 min. Pri-miRNA and mCherry expression was induced via addition of 1 µg/mL doxycycline (Clontech). Image acquisition and analysis was performed using MetaMorph software (Universal Imaging Corporation). The fluorescence intensities of GFP, dsGFP, dsCFP, and dsVenus were quantified in mCherry-expressing cells. When cells underwent cell division during imaging, the average intensity of the two daughter cells was analyzed. To determine protein turnover of miRNA sensors, stable HeLa cells expressing miRNA sensors were added with 100 µg/mL CHX or 0.1% DMSO. For imaging neurons, primary cortical neurons plated in 35-mm glass bottom dishes (DIV 2-5) were transfected with dsVenus/dsCFP or dsVenus-132-T/dsCFP. The next day, the cells were processed for time-lapse imaging as described above. miR-132-3p expression was induced via addition of 50 ng/mL brain-derived neurotrophic factor (BDNF; Millipore).

### Mathematical modeling

Detailed procedures for mathematical modeling are described in Supplementary methods.

### Statistical analysis

Statistical significance was analyzed using Kruskal-Wallis test followed by Steel-Dwass test, Spearman’s correlation coefficient, or two-way repeated measures ANOVA with multiple comparisons. *p < 0.05, **p < 0.01.

### Data availability

All data generated or analysed during this study are included in this published article and its Supplementary Information files.

## Electronic supplementary material

Supplementary Information
