# Supplementary material for: Time-lapse imaging of microRNA activity reveals the kinetics of microRNA activation in single living cells
Source: Sci Rep. 2017 Oct 3;7:12642. doi: 10.1038/s41598-017-12879-2 (PMC5626736; doi:10.1038/s41598-017-12879-2)
Supplement: Supplementary file 1 — Supplementary Information [file 41598_2017_12879_MOESM1_ESM.pdf]

Supplementary Information

Time-lapse imaging of microRNA activity reveals the kinetics of microRNA activation in single living cells

Hideaki Ando, Matsumi Hirose, Gen Kurosawa, Soren Impey, and Katsuhiko Mikoshiba

Supplementary methods

Mathematical modeling

The dynamics of miRNAs and miRNA targets were modeled as follows:

$$dX/dt = f - aX \quad (1)$$

$$dY/dt = q - v(1 + l\,X^n/(K_M + X^n))Y \quad (2)$$

$$dZ/dt = sY/(K_I + X^m) - dZ \quad (3)$$

where the abundance of the miRNA, target mRNA, and protein are expressed as *X, Y,* and *Z*, respectively. On the right-hand side of the equations, the first term represents the synthesis, and the second term represents the degradation of each variable. The synthesis of the miRNA and target mRNA were assumed to occur with constant speed (*f* and *q*, respectively). Thus, *q* is the transcription speed and *v* is the decay rate of target mRNA in the absence of miRNA. The up-regulation of target mRNA degradation and the down-regulation of translation by miRNA were expressed by the nonlinear functions $X^n/(K_M + X^n)$ and $1/(K_I + X^m)$ of the miRNA (*X*), respectively (65). The other terms were expressed by a linear function of the substrate, for simplicity. In the time-lapse imaging experiments, the transcription of pri-miRNA was induced at *t* = 4 h, and then, the levels of the target mRNA and protein decreased from the basal levels. To reproduce the experimental time series (Fig. 2f, 3e), miRNA level (*X*) was fixed at 0, and levels of both target mRNA and protein were fixed at basal levels before miRNA induction. After miRNA induction (*t* = 4 h), pri-miRNA transcription commenced. As miRNA (*X*) accumulated, it up-regulated the degradation of target mRNA (*Y*), and/or down-regulated translation, which led to a decrease in the levels of the target protein (*Z*). The equations were solved numerically using the Euler Method with delta *t* = 0.01.

For certain parameter sets, the experimental time series of the target protein (*Z*) (Fig. 2f, 3e) could be realized. First, we obtained the degradation rates (*d*) of the target proteins dsGFP-138-T and dsGFP-295-T from their experimentally measured half-lives. Since both the target proteins remained at basal levels before miRNA induction (*t* = 0~4 h, Fig. 2f, 3e), the translation rate (s/K_I) in the absence of miRNA regulation (*m* = 0) could be determined. Second, we computationally

searched for the parameter set for the dynamics of the miRNA (*X*) and target mRNA (*Y*), which reproduced the experimental data of the time series of the miRNA (Fig. 4b-d, red dots) and target mRNA (Fig. 4b-d, orange dots). Finally, if the miRNA regulated the target mRNA only through mRNA degradation (no translation regulation, $m = 0$), the time series of the target protein (Fig. 2f, 3e) should be realized using the parameters that reproduced the time series of the target mRNA and the parameters of the dynamics of the target protein (d and s/K_I). Indeed, the time series of the target proteins, dsGFP-138-T, under pri-miR-138-1 induction (Fig. 2f) and dsGFP-295-T, under pri-miR-294/295 induction (Fig. 3e), were successfully realized without further parameter tuning (Fig. 4b, 4c). In contrast, in order to reproduce the time series of dsGFP-295-T under pri-miR-294/295mut induction (Fig. 3e), translational regulation by miRNA was required ($m > 0$). The parameter for translational regulation was tuned in order to reproduce the data ($m = 2$) (Fig. 4d). The parameters for the dynamics of dsGFP-138-T under pri-miR-138-1 induction were as follows: $d = 0.408$, $s = 0.816$, $K_I = 1$, $f = 0.099$, $a = 0.088$, $q = 5$, $v = 5$, $l = 3$, $K_M = 0.05$, $n = 4$, and $m = 0$ (Fig. 4b). The parameters for the dynamics of dsGFP-295-T under pri-miR-294/295 induction were as follows: $d = 0.3$, $s = 0.6$, $K_I = 1$, $f = 0.14$, $a = 0.135$, $q = 5$, $v = 5$, $l = 170$, $K_M = 35$, $n = 2$, and $m = 0$ (Fig. 4c). The parameters for the dynamics of dsGFP-295-T under pri-miR-294/295mut induction were as follows: $d = 0.3$, $s = 0.03$, $K_I = 0.1$, $f = 0.068$, $a = 0.048$, $q = 5$, $v = 5$, $l = 110$, $K_M = 35$, $n = 4$, and $m = 2$ (Fig. 4d).

To use the data of the experimental time series for parameter fitting, the abundance of the miRNA, target mRNA, and protein was normalized. The abundance of miRNA (*X*) at t = 24 h, and of both target mRNA (*Y*) and protein (*Z*) at $t = 0$ was normalized to unity. It is noteworthy that the co-transfection rate of the miRNA plasmid and the tTA plasmid was 36.9% for mCherry/pri-miR-138-1, 30.5% for mCherry/pri-miR-294/295, and 41.3% for mCherry/pri-miR-294/295mut, respectively (Supplementary Fig. S3 online), which implied that certain cells with the miRNA target in the population did not contain the miRNA or tTA plasmid. Our model assumed that all the cells with the miRNA target contained the miRNA and tTA plasmids. Thus, we modified the time series of the target mRNA under pri-miRNA induction (Fig. 2h, 3h), by which the decrease in the target mRNA level from the basal level was multiplied by the inverse of the transfection rate. When the mRNA levels had negative values, these time points (24 h) were omitted. This might have been caused by the lower rates of mCherry expression, with levels detectable by fluorescence microscopy, than the rates of miRNA expression.

References

65. Murray, J. D. *Mathematical Biology: I. Introduction* 175-217 (Springer, 2002).

**miRNA target sequences**		
miR-138-5p-target	sense	5'-*TCGAC*CGGCCTGATTCACAACACCAGCTAGATCTCGGCCTGATTCACAACACCAGCT*G*-3'
(for pdsGFP)	antisense	5'-*GATCC*AGCTGGTGTTGTGAATCAGGCCGAGATCTAGCTGGTGTTGTGAATCAGGCCG*G*-3'
miR-138-5p-mut	sense	5'-*TCGAC*CGGCCTGATTCACAACTGGTCGTAGATCTCGGCCTGATTCACAACTGGTCGT*G*-3'
(for pdsGFP)	antisense	5'-*GATCC*ACGACCAGTTGTGAATCAGGCCGAGATCTACGACCAGTTGTGAATCAGGCCG*G*-3'
miR-295-3p-target	sense	5'-*TCGAC*AGACTCAAAAGTAGTAGCACTTTAGATCTAGACTCAAAAGTAGTAGCACTTT*G*-3'
(for pdsGFP)	antisense	5'-*GATCC*AAAGTGCTACTACTTTTGAGTCTAGATCTAAAGTGCTACTACTTTTGAGTCT*G*-3'
miR-295-3p-mut	sense	5'-*TCGAC*AGACTCAAAAGTAGTACGTTACTAGATCTAGACTCAAAAGTAGTACGTTACT*G*-3'
(for pdsGFP)	antisense	5'-*GATCC*AGTAACGTACTACTTTTGAGTCTAGATCTAGTAACGTACTACTTTTGAGTCT*G*-3'
miR-9-5p-target	sense	5'-*GATCC*TCATACAGCTAGATAACCAAAGAAGATCTTCATACAGCTAGATAACCAAAGA*C*-3'
(for pdsVenus)	antisense	5'-*TCGAG*TCTTTGGTTATCTAGCTGTATGAAGATCTTCTTTGGTTATCTAGCTGTATGA*G*-3'
miR-9-3p-target	sense	5'-*TCGAC*ACTTTCGGTTATCTAGCTTTATAGATCTACTTTCGGTTATCTAGCTTTAT*G*-3'
(for pdsCFP)	antisense	5'-*GATCC*ATAAAGCTAGATAACCGAAAGTAGATCTATAAAGCTAGATAACCGAAAGT*G*-3'
miR-132-3p-target	sense	5'-*TCGAC*CGACCATGGCTGTAGACTGTTAAGATCTCGACCATGGCTGTAGACTGTTA*G*-3'
(for pdsGFP)	antisense	5'-*GATCC*TAACAGTCTACAGCCATGGTCGAGATCTTAACAGTCTACAGCCATGGTCG*G*-3'
(for pdsVenus)	sense	5'-*GATCC*CGACCATGGCTGTAGACTGTTAAGATCTCGACCATGGCTGTAGACTGTTA*C*-3'
	antisense	5'-*TCGAG*TAACAGTCTACAGCCATGGTCGAGATCTTAACAGTCTACAGCCATGGTCG*G*-3'
miR-132-3p-mut	sense	5'-*TCGAC*CGACCATGGCTGTAGTGGACCAAGATCTCGACCATGGCTGTAGTGGACCA*G*-3'
(for pdsGFP)	antisense	5'-*GATCC*TGGTCCACTACAGCCATGGTCGAGATCTTGGTCCACTACAGCCATGGTCG*G*-3'
**Cloning of pri-miRNAs**		
pri-miR-138-1	forward	5'-AAA*GGTACC*CCTTACTCCTCACTGTCATC-3'
	reverse	5'-AAA*GCGGCCGC*TTTCCTCACAGGCAGGTGCTC-3'
pri-miR-294/295	forward	5'-AAA*GGTACC*TGAGGTGCACGGAACCTCAC-3'
	reverse	5'-AAA*GTCGAC*GGCACTGGTTGCTCCCATAG-3'
pri-miR-132	forward	5'-AAA*GGTACC*TGCTGATGCAGTGCAAGCGC-3'
	reverse	5'-AAA*AAGCTT*AGTCTCTGAGGAGGATGTTC-3'
pri-miR-9-1	forward	5'-AAA*GGTACC*CTCGCCTTTCTGAGGTCTCG-3'
	reverse	5'-AAA*GCGGCCGC*CTTCCCAGGTGAGCAGGTTG-3'
**Quantitative RT-PCR**		
GFP/dsGFP	forward	5'-CGACCACTACCAGCAGAACACC-3'
	reverse	5'-ACTCCAGCAGGACCATGTGATC-3'
GAPDH	forward	5'-AATCCCATCACCATCTTCCA-3'
	reverse	5'-TGGACTCCACGACGTACTCA-3'
**Synthetic RNA**		
miR-138-5p		5'-AGCUGGUGUUGUGAAUCAGGCCG-3'
miR-294-3p		5'-AAAGUGCUUCCCUUUUGUGUGU-3'
miR-295-3p		5'-AAAGUGCUACUACUUUUGAGUCU-3'
miR-9-5p		5'-UCUUUGGUUAUCUAGCUGUAUGA-3'
miR-9-3p		5'-AUAAAGCUAGAUAACCGAAAGU-3'
miR-132-3p		5'-UAACAGUCUACAGCCAUGGUCG-3'

Supplementary Table S1. Primers and oligonucleotides.

miRNA target sequences are underlined. Mutation sites are indicated by red font.

Restriction enzyme sites are shown in italic.

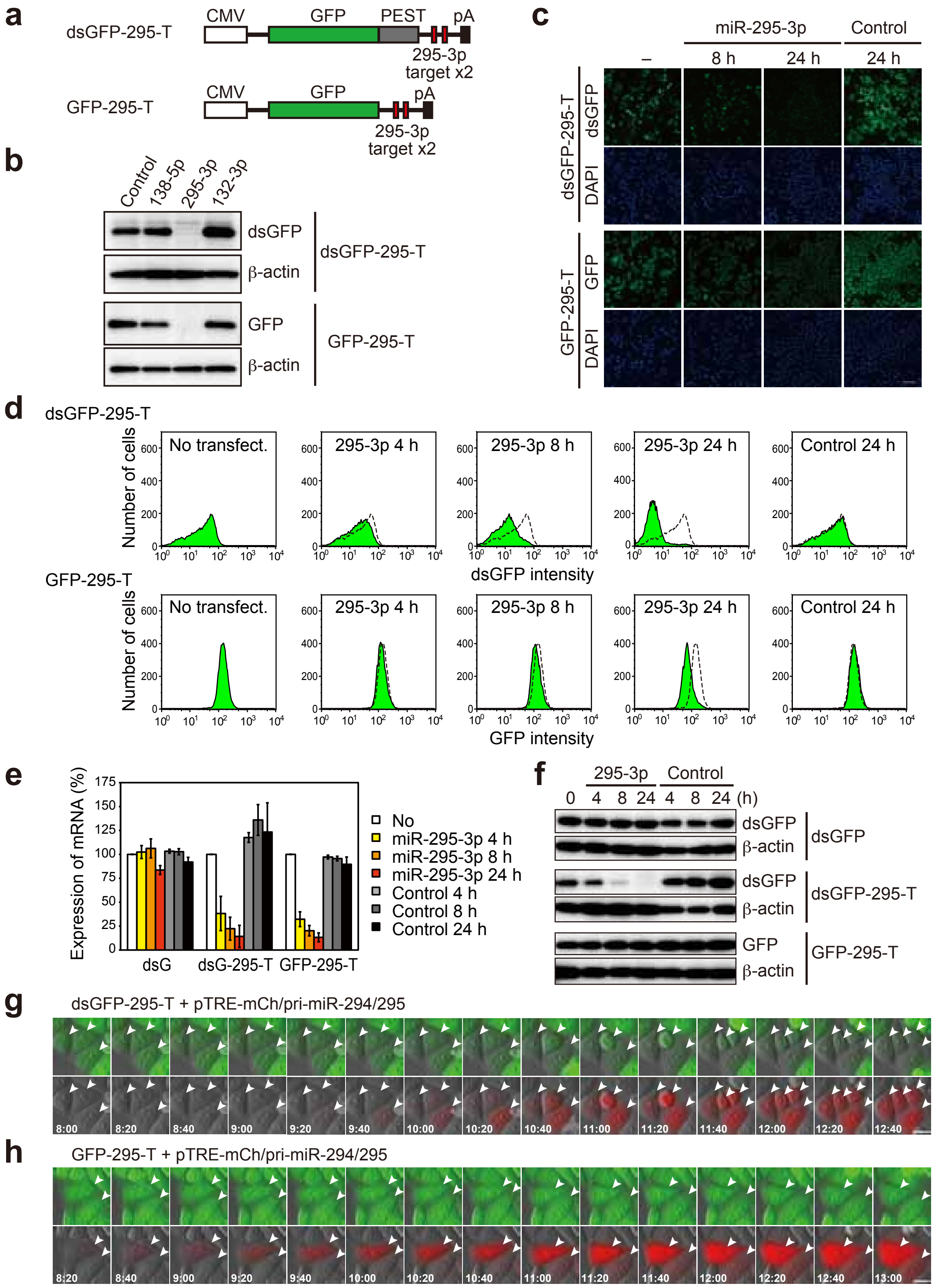

Supplementary Figure S1. Characterization of miR-295-3p sensors.
(a) Structure of miR-295-3p sensors. Two binding sites of miR-295-3p were inserted in the 3′ UTR of dsGFP or GFP. (b) Plasmids encoding dsGFP-295-T or GFP-295-T (0.5 µg) were co-transfected with 1 nM miR-138-5p, miR-295-3p, miR-132-3p, or control miRNA mimics into HeLa cells. 24 h after transfection, cells were analyzed by western blotting with anti-GFP and anti-β-actin antibodies. (c, d) Stable HeLa cells expressing dsGFP-295-T or GFP-295-T were transfected with 1 nM miR-295-3p or control miRNA mimics. Fluorescence of miRNA sensors was analyzed by confocal microscopy (c) or flow cytometry (d). Scale bars: 100 µm. (e) Stable HeLa cells expressing dsGFP, dsGFP-295-T, or GFP-295-T were transfected with 1 nM miR-295-3p or control miRNA mimics. mRNA expression levels of miRNA sensors were analyzed by quantitative RT-PCR. Data are expressed as mean ± SD (n = 3). (f) Stable HeLa cells transfected with miRNA mimics were analyzed by western blotting with anti-GFP and anti-β-actin antibodies. (g, h) Stable HeLa cells expressing dsGFP-295-T (g) or GFP-295-T (h) were transfected with pTRE-mCh/pri-miR-294/295 and tTA, and the expression of pri-miR-294/295 and mCherry were induced by doxycycline. Fluorescence of miR-295-3p sensors and mCherry were captured every 20 min at 37 ° C. mCherry-expressing cells are indicated by arrowheads. Scale bars: 20 µm.

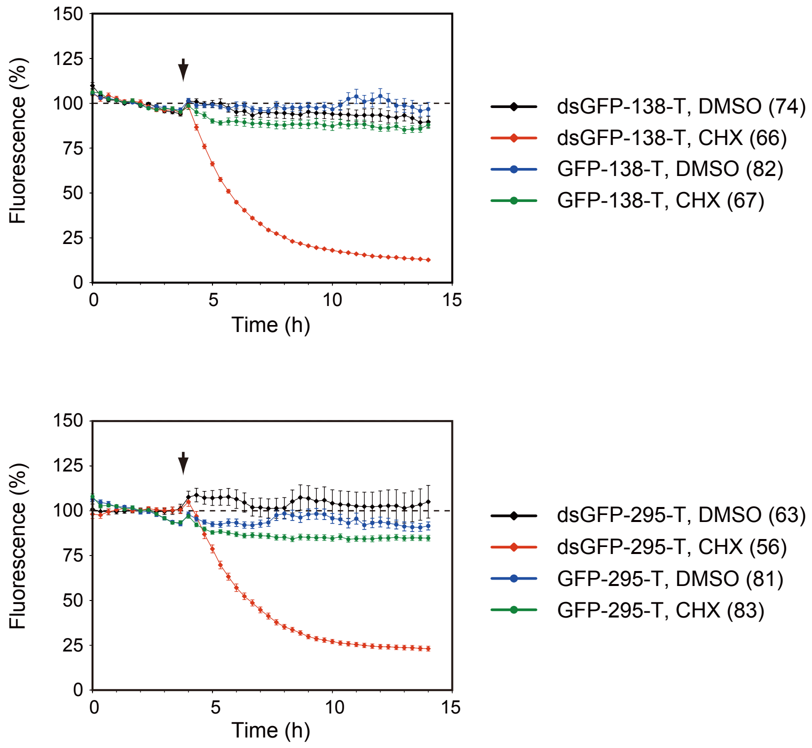

Supplementary Figure S2. Decay kinetics of miRNA sensors by CHX treatment.
Stable HeLa cells expressing miRNA sensors were treated with 100 μg/mL CHX or 0.1% DMSO at the indicated time. Fluorescence intensities are presented as mean± SEM. Numbers of cells analyzed are shown in parentheses.

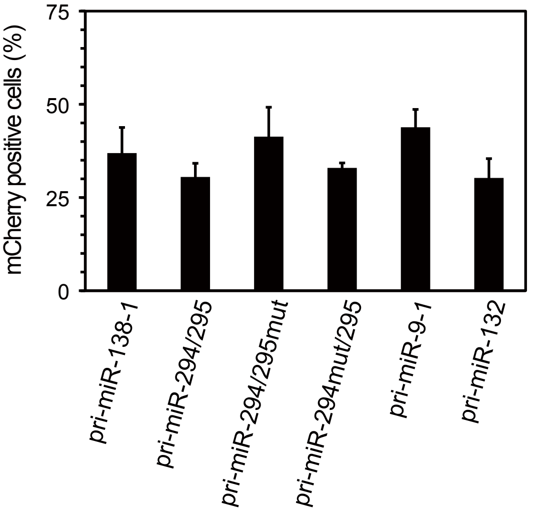

Supplementary Figure S3. Co-transfection efficiency of mCherry/pri-miRNA and tTA expression vectors. The pTRE-mCherry/pri-miRNA vector and the tTA expression vector were transfected into stable HeLa cells and expression of mCherry and pri-miRNA were induced by 1 μg/mL doxycycline for 24 h. Percentage of mCherry positive cells were counted. Data are expressed as mean ± SD (n = 3).

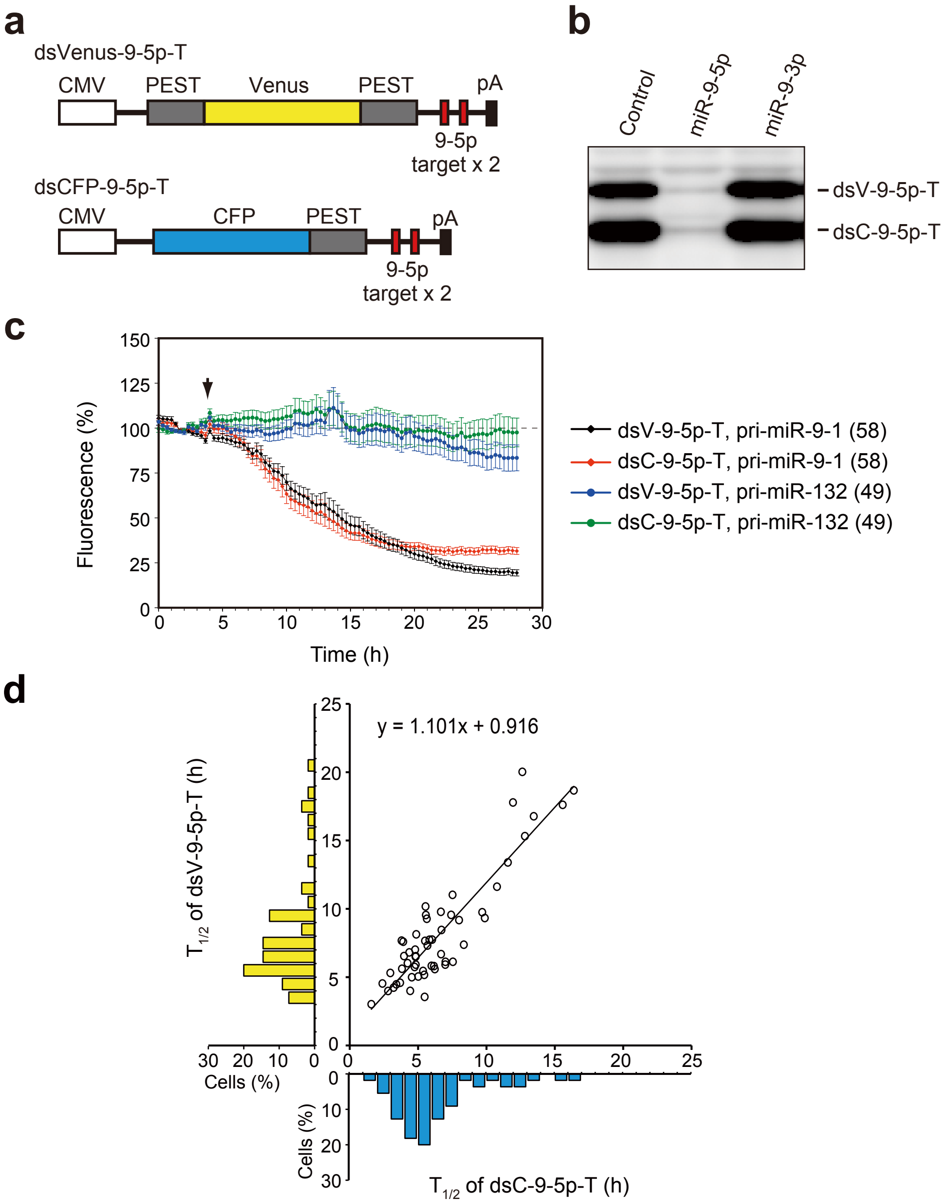

Supplementary Figure S4. Dual imaging of dsVenus-9-5p-T and dsCFP-9-5p-T.
(a) dsVenus-9-5p-T and dsCFP-9-5p-T sensors. (b) Stable HeLa cells expressing both dsVenus-9-5p-T and dsCFP-9-5p-T were transfected with 1 nM miR-9-5p, miR-9-3p, or control miRNA mimics. 24 h after transfection, cells were analyzed by western blotting with anti-GFP antibody. (c) Stable HeLa cells expressing both dsVenus-9-5p-T and dsCFP-9-5p-T were transfected with pTRE-mCh/pri-miR-9-1 and tTA, and expression of pri-miR-9-1 was induced by doxycycline. Relative fluorescence intensities of dsVenus-9-5p-T and dsCFP-9-5p-T are presented as mean ± SEM. Numbers of cells analyzed are shown in parentheses. (d) T1/2 of dsCFP-9-5p-T (x-axis) and dsVenus-9-5p-T (y-axis) in individual cells are plotted (55 cells). Marginal histograms show distributions of T1/2 of dsCFP-9-5p-T (bottom) and dsVenus-9-5p-T (left).

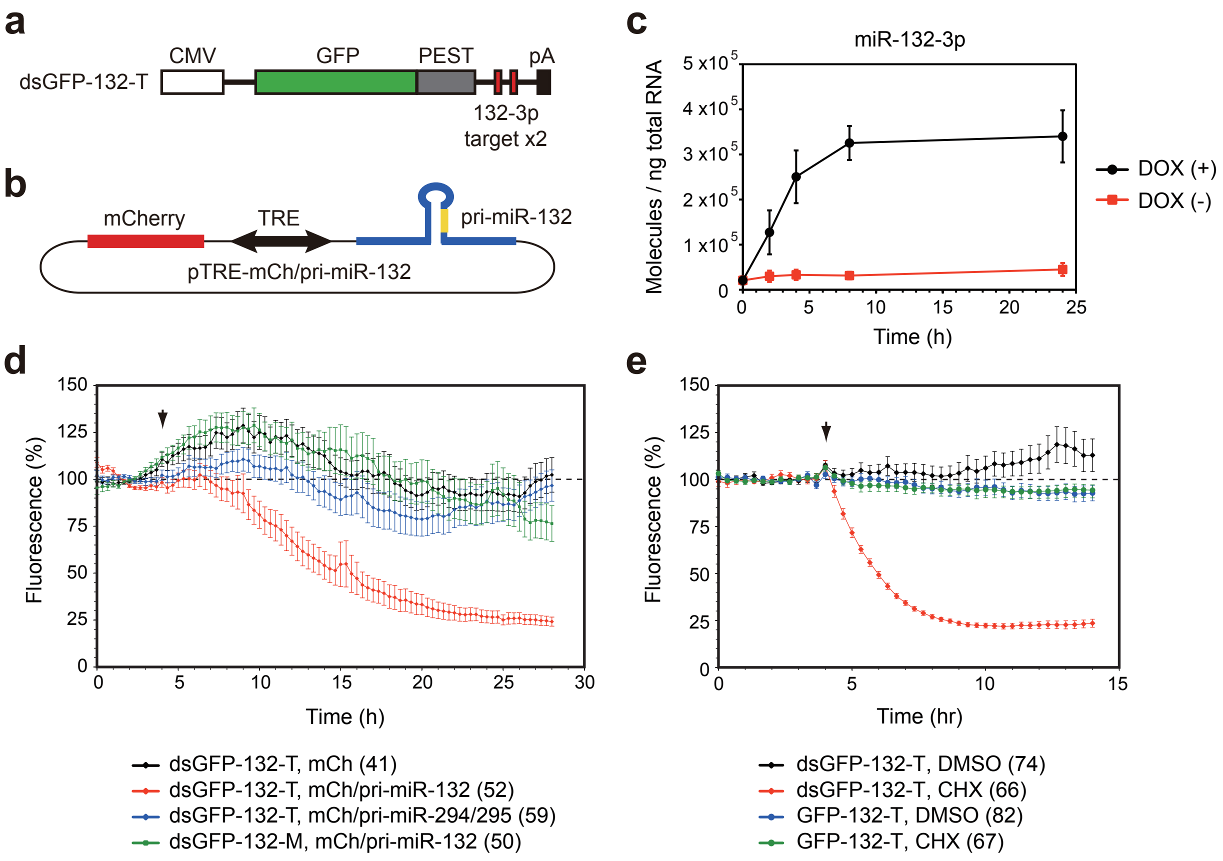

Supplementary Figure S5. Time-lapse imaging of the miR-132-3p sensor in living cells.
(a) miR-132-3p sensor. (b) pTRE-mCh/pri-miR-132 vector. (c) Stable HeLa cells expressing dsGFP-132-T were transfected with pTRE-mCh/pri-miR-132 and tTA. The next day, the cells were incubated with or without 1 μg/mL doxycycline for 2, 4, 8 or 24 h. Total RNA was purified and expression of miR-132-3p was analyzed by quantitative RT-PCR. Data are expressed as mean ± SD (n = 3). (d) Stable HeLa cells expressing dsGFP-132-T or dsGFP-132-M were transfected with the pTRE-mCh/pri-miRNA expression vector and tTA, and expression of pri-miRNAs were induced by doxycycline. Relative fluorescence intensities of dsGFP-132-T and dsGFP-132-M are presented as mean ± SEM. Numbers of cells analyzed are shown in parentheses. (e) Stable HeLa cells expressing dsGFP-132-T or GFP-132-T were treated with 100 μg/mL CHX or 0.1% DMSO at the indicated time. Fluorescence intensities are presented as mean ± SEM. Numbers of cells analyzed are shown in parentheses.

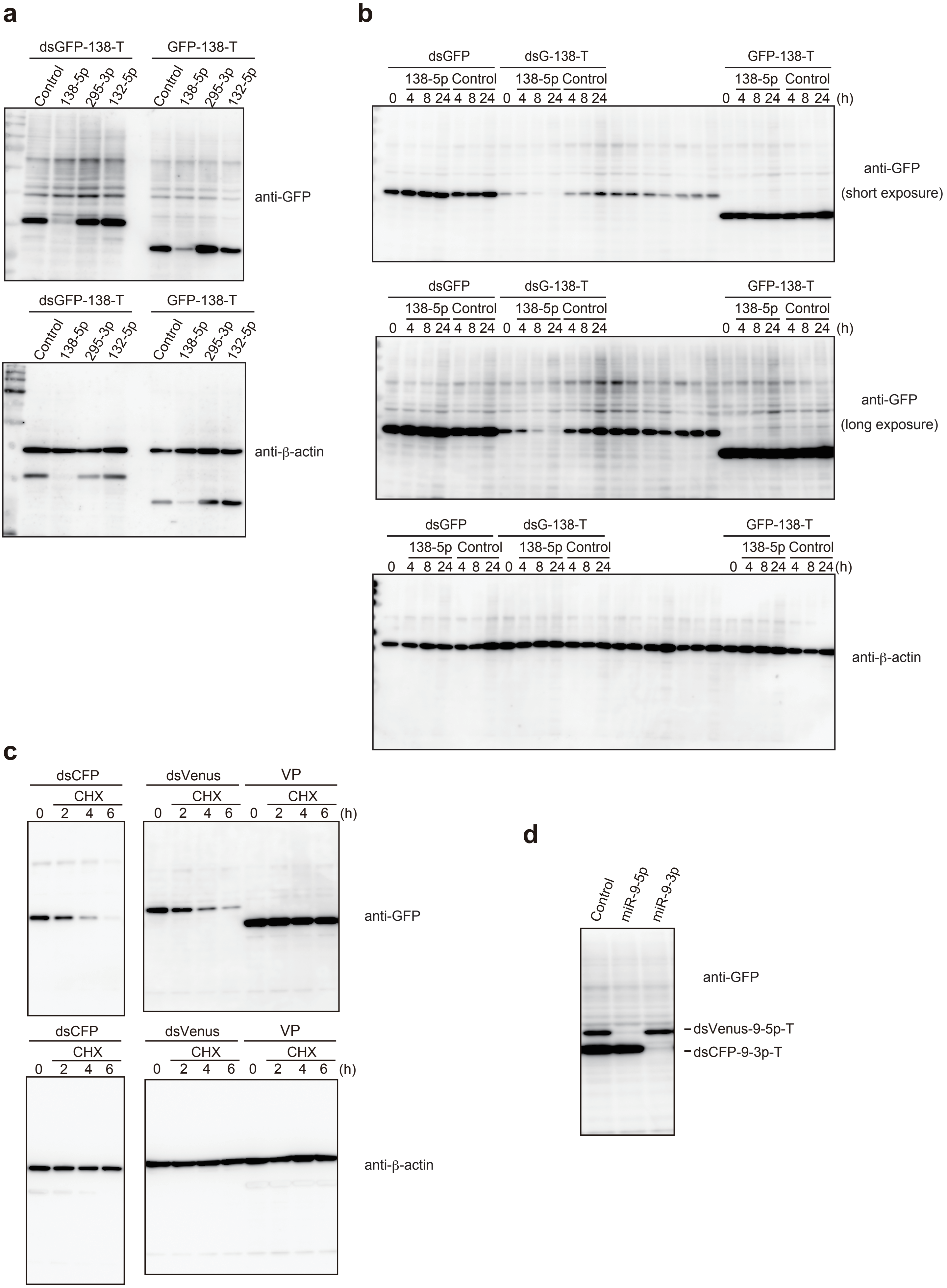

Supplementary Figure S6. Full-length blots of western blotting data in Fig. 1b (a), Fig. 1f (b), Fig. 5a (c), and Fig. 5c (d).
